# Dynamics of Scabin toxin. A proposal for the binding mode of the DNA substrate

**DOI:** 10.1371/journal.pone.0194425

**Published:** 2018-03-15

**Authors:** Miguel R. Lugo, Bronwyn Lyons, Cristina Lento, Derek J. Wilson, A. Rod Merrill

**Affiliations:** 1 Department of Molecular and Cell Biology, University of Guelph, Guelph, Ontario, Canada; 2 Department of Biochemistry and Molecular Biology, University of British Columbia, Vancouver, British Columbia, Canada; 3 Chemistry Department, York University, Toronto, Ontario, Canada; 4 The Centre for Research in Mass Spectrometry, York University, Toronto, Ontario, Canada; Weizmann Institute of Science, ISRAEL

## Abstract

Scabin is a mono-ADP-ribosyltransferase enzyme and is a putative virulence factor produced by the plant pathogen, *Streptomyces scabies*. Previously, crystal structures of Scabin were solved in the presence and absence of substrate analogues and inhibitors. Herein, experimental (hydrogen-deuterium exchange), simulated (molecular dynamics), and theoretical (Gaussian Network Modeling) approaches were systematically applied to study the dynamics of apo-Scabin in the context of a Scabin·NAD^+^·DNA model. MD simulations revealed that the apo-Scabin solution conformation correlates well with the X-ray crystal structure, beyond the conformation of the exposed, mobile regions. In turn, the MD fluctuations correspond with the crystallographic B-factors, with the fluctuations derived from a Gaussian network model, and with the experimental H/D exchange rates. An Essential Dynamics Analysis identified the dynamic aspects of the toxin as a crab-claw-like mechanism of two topological domains, along with coupled deformations of exposed motifs. The “crab-claw” movement resembles the motion of C3-like toxins and emerges as a property of the central β scaffold of catalytic single domain toxins. The exposure and high mobility of the *cis* side motifs in the Scabin β-core suggest involvement in DNA substrate binding. A ternary Scabin·NAD^+^·DNA model was produced via an independent docking methodology, where the intermolecular interactions correspond to the region of high mobility identified by dynamics analyses and agree with binding and kinetic data reported for wild-type and Scabin variants. Based on data for the Pierisin-like toxin group, the sequence motif R_β1_–R_La_–N_Lc_–STT_β2_–W_PN_–W_ARTT_–(QxE)_ARTT_ emerges as a catalytic signature involved in the enzymatic activity of these DNA-acting toxins. However, these results also show that Scabin possesses a unique DNA-binding motif within the Pierisin-like toxin group.

## Introduction

Bacterial mono-ADP-ribosyltransferase (mART) toxins comprise an important class of virulence factors produced by bacterial pathogens[1]. The cellular targets for mART toxins are often key regulators of cell function and include (i) GTPases, (ii) actin, (iii) kinase regulators, (iv) elongation factors, (v) RNA-recognition motifs, and even DNA (target genes) [2]. These mART toxins are enzymes that bind NAD^+^, cleave its glycosidic bond (C-N), and transfer ADP-ribose to a target macromolecule (usually protein). In addition, mART toxins possess glycohydrolase activity (GH) that has no known biological function [3]. The covalent modification of the target macromolecule (ADP-ribosylation) by mART toxins alters its function and can act as an “on”–“off” switch for activity [1].

S*treptomyces scabies* is a filamentous, soil-dwelling plant pathogen [4]. *S*. *scabies* is the causative agent of the common scab disease that affects taproot and tuberous vegetables, producing deep-pitted and corky lesions on the surface of the tuber, impacting the market value of the infected crop [5, 6]. The common scab disease is of global economic importance, as there is currently no effective pesticide treatment once a field is contaminated with the pathogen. Recently, the use of biocontrol agents has been studied to help suppress *S*. *scabies* growth and reduce the progression of the common scab disease [7, 8].

Scabin is a 200-residue, 22-kDa, single-domain enzyme produced and secreted by *S*. *scabies* [9, 10]. Scabin was cloned, purified and shown to possess both GH and ADP-ribosyltransferase activities. Scabin is one of only a few members of the mART toxin family that utilizes DNA as a target macromolecular substrate, as well as exhibiting specificity towards genomic DNA from potato tubers (*Solanum tuberosum*). Previous bioinformatic analyses revealed that Scabin shares nearly 40% sequence identity with the Pierisin family of eukaryotic mART toxins [9]. The Pierisin family is distinguished by its unique target specificity where ADP-ribose is transferred to the guanine base in DNA, leading to host-cell apoptosis from *Pieris rapae* [11, 12]. Scabin therefore represents the first mART of bacterial origin that labels DNA as its target substrate [9].

A bacterial toxin/enzyme that modifies DNA has been recently identified and is known as DarT [13]. DarT is an enzyme that specifically modifies the second thymidine base in the TNTC motif of ssDNA with ADP-ribose. This ADP-ribose modification of DNA can be removed by the DNA ADP-ribose glycohydrolase, DarG, which is a macrodomain protein. This constitutes a novel DNA-ribosylating toxin-antitoxin system that is present in a variety of bacterial species, including human pathogens [2]. The mechanism and structure of the DarT enzyme awaits further characterization.

Kinetic characterization of Scabin revealed a highly active enzyme (GH activity) when compared to other members of the mART family. Notably, Scabin exhibited sigmoidal kinetic behavior in the presence of the deoxyguanosine substrate, unlike the Michaelis-Menten behavior of most mART toxins. In our earlier report, we presented the first crystal structure of Scabin as a DNA-acting mART, as well as co-crystal structures of two good (lead) inhibitors of Scabin activity. A working model was developed of the Scabin·NAD^+^·DNA complex to help guide future experiments, including mutagenesis of the active-site architecture [9].

Recently, we investigated the role of several catalytic residues in Scabin that participate in DNA binding and enzyme function [14] We solved the crystal structure of Scabin with NADH, which is a potent competitive inhibitor against the NAD^+^ substrate. This complex was used to shed important insights into the nature of the Scabin·NAD^+^ structure and to assist in the interpretation of the kinetic experiments involving Scabin catalytic variants. We characterized the transferase activity and binding of Scabin for the DNA substrate. Notably, Scabin exhibited a modest increase in affinity for double-stranded DNA containing a single base overhang, when compared to single or blunt-ended double-stranded substrate. Based on the observed binding and kinetic data involving the wild-type and catalytic variants, a DNA-binding mechanism for Scabin was proposed, representing the first evidence of a DNA-binding motif for bacterial mART toxins [14].

The Scabin toxin crystal structure was previously reported for the enzyme in the presence and absence of NAD^+^ competitive inhibitors [9]. The Scabin structure displays a characteristic mART fold and contains the conserved R-S-Q-X-E motif. Scabin shows only ~32% α/β structure and unlike other mART enzymes is largely dominated by coiled structure, although it is highly ordered [9]. Furthermore, Scabin has low sequence identity with most mART toxins except for the Pierisin subgroup and significantly differs in topology from well characterized mART toxins like *iota* and C3-group members, which are dominated by high helical content at the N-terminus [15]. Scabin shares structural homology with Mosquitocidal toxin (MTX) from *Bacillus sphaericus* [16] and the apoptosis-inducing Pierisin-1 from the cabbage butterfly *Pieris rapae* [17]. The roles of the ARTT and PN loops are well defined in mART toxins [18, 19]. However, the location and topology of the α_1_-α_2_ motif is unique for the Pierisin-like subgroup of the CT group of mART toxins with known structures [9, 17]. Thus, it is important to consider the role of the α_1_-α_2_ motif in the dynamic and catalytic properties of Pierisin-like toxins beyond its obvious structural role.

In this work, experimental (hydrogen-deuterium exchange, HDX), simulated (molecular dynamics, MD), and theoretical (Gaussian Network Modeling, GNM) [20, 21] approaches were systematically applied to study the dynamics of apo-Scabin in the context of a Scabin·NAD^+^·DNA model. HDX measurements on Scabin toxin in the presence and absence of substrates were conducted to provide new insights into the solution dynamics of the enzyme and its substrate-binding “footprint”. MD simulations were used to compare the apo-Scabin solution conformation with the X-ray crystal structure. GNM and Essential Dynamics Analysis (EDA) were used to produce a dynamic profile of the toxin with its crab-claw-like mechanism of two topological domains. The “crab-claw” motion resembles the motions of C3-like toxins and is an intrinsic conformational property of the central β-scaffold of catalytic single-domain toxins. The exposure and high mobility of the motifs at the *cis* side of the Scabin β-core suggest involvement in DNA substrate binding. A novel, ternary Scabin·NAD^+^·DNA model was produced via an independent docking methodology, where the nature of the intermolecular interactions is separated into an “electrostatic” *lower-area* and “hydrophobic” *upper-area*. The *middle-area* interactions are mixed in character with both NAD^+^ and DNA substrates bridged by the participating residues, including Tyr129 which plays a major role in DNA binding. Based on data for the Pierisin-like toxin group, the sequence motif R_β1_−R_La_−N_Lc_−STT_β2_−W_PN_−W_ARTT_−(QxE)_ARTT_ emerges as a catalytic signature involved in the enzymatic activity of these DNA-acting toxins. However, these results suggest that Scabin possesses a unique DNA-binding motif within the Pierisin-like toxin group.

## Results

**Fig 1:**
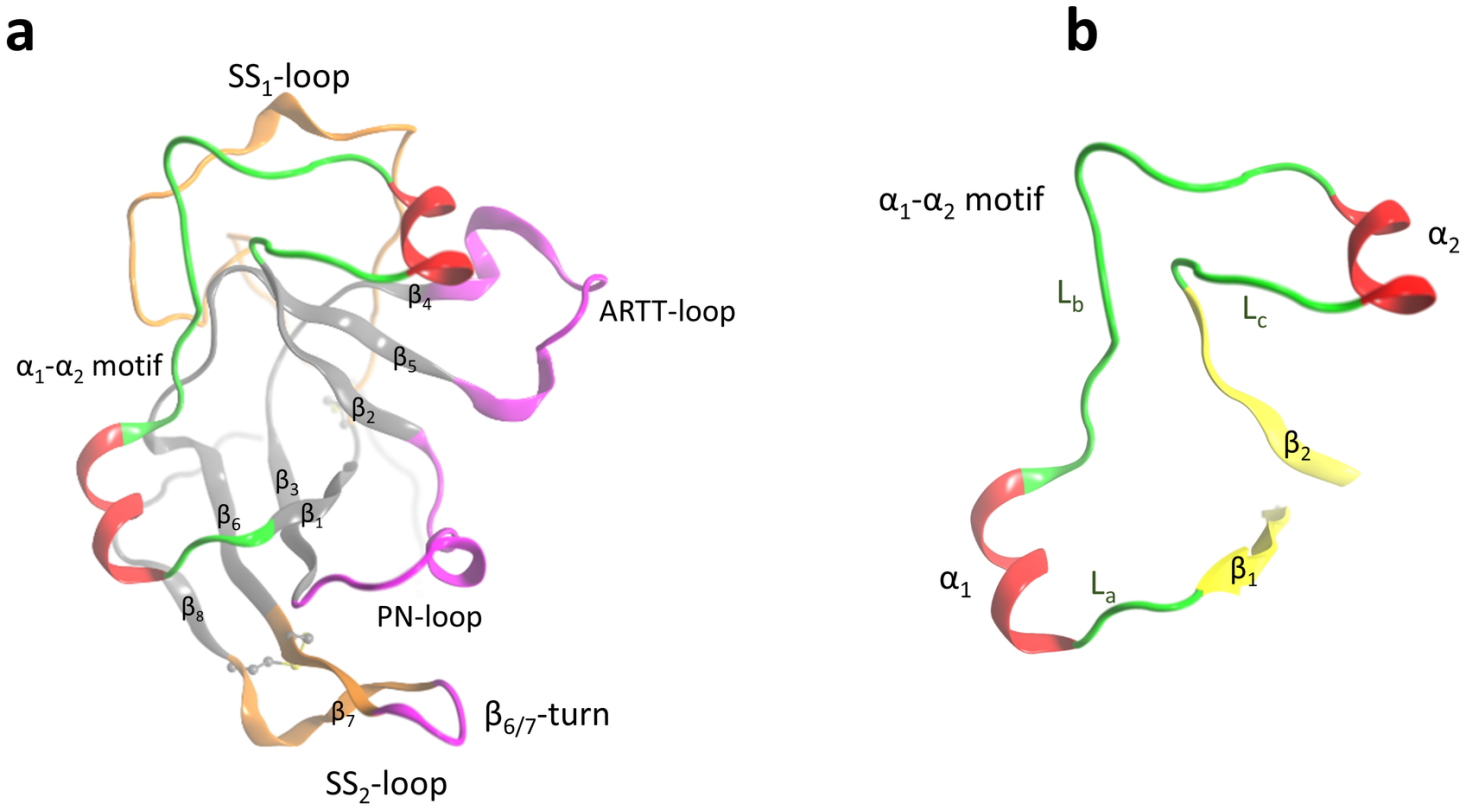
Scabin 3D-topology and sequence. (**a**) Ribbon representation of Scabin X-ray structure (5DAZ) with sequential nomenclature, α_i_ for helical and β_i_ for β-strand secondary-structure elements. The PN loop (Y120-G132), ARTT loop (V145-E160), and β_6/7_-turn (K180-R184) are depicted in fuchsia, the SS_1_-loop (C42-C72) and the SS_2_-loop (C176-C190) in amber, and the α_1_-α_2_ motif (S80-P114) is highlighted with the α-helices in red and coiled segments in green. (**b**) The isolated α_1_-α_2_ motif flanked by β_1_ and β_2_ shows the nomenclature for the secondary-structure elements as: L_a_ (S80-G82), α_1_ (P83-Q89), L_b_ (G90-D102), α_2_ (I103-V109), and L_c_ (N10-P114).

The crystal structure of Scabin was solved to 1.5 Å resolution in the apo-form by Lyons *et al*. (2016) [9] and deposited in the Protein Data Bank (PDB) with the code 5DAZ. Scabin possesses a mixed α/β-fold with a central β-sheet core formed by a four-stranded β_I_-sheet (β_1_, β_3_, β_6_, and β_7_-β_8_) in a perpendicular arrangement to a three-stranded β_II_-sheet (β_2_, β_4_, and β_5_), typically seen for the β-fold of catalytic domains of mART toxins (Fig 1a). Facing the NAD^+^-binding pocket (*cis* side), the ARTT-loop (ADP-ribosyl-turn-turn loop) links β_4_ with β_5_ within the β_I_-sheet, and the β_6/7_-turn links β_6_ with β_7_-β_8_ within the β_II_-sheet. In addition, two other *cis* segments connect the two β-sheets: the PN-loop (phosphate-nicotinamide loop) that links β_2_ with β_3_, and the α_1_-α_2_ motif that links β_1_ with β_2_. The α_1_-α_2_ motif consists of coiled (L) and helical (α) structural elements that alternate according to the L_a_-α_1_-L_b_-α_2_-L_c_ pattern (Fig 1b).

### I. Essential dynamics of apo-Scabin

**Fig 2:**
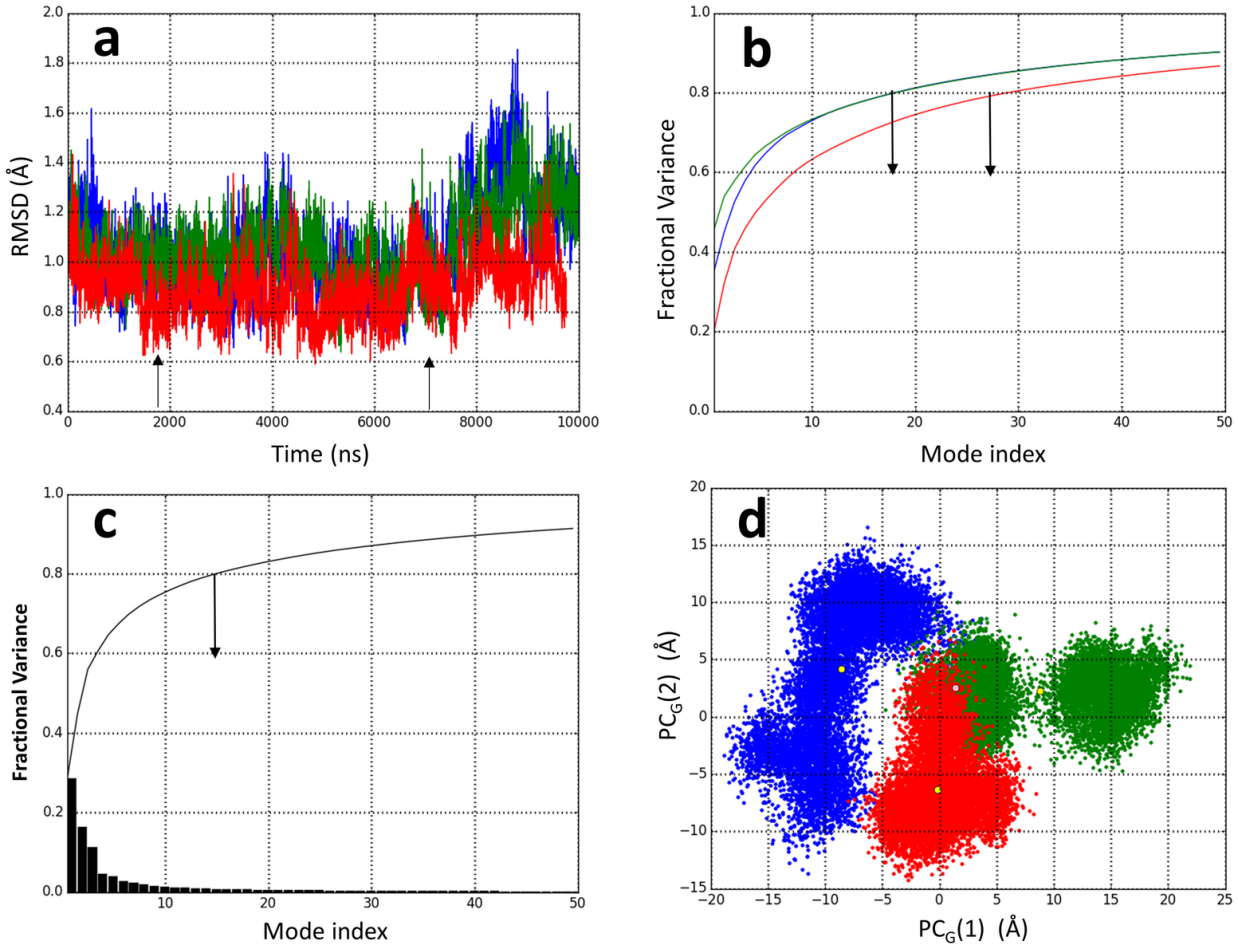
Apo-Scabin MD trajectories and EDA. (**a**) RMDS time-course of 10,000 interposed configurations of the C_α_-atoms of apo-Scabin from three 100 ns MD simulations, colored by trajectory as Run_a_ in blue, Run_b_ in green, and Run_c_ in red. The region confined by the arrows represents the period when the trajectories were relatively constant in the plotted variable. (**b**) Cumulative fractional variance for the first 50 PC modes from an EDA for each of the ensembles of MD configurations as colored in (a). The arrows point to the mode index in which the cumulative fractional variance is 0.8 in each case. (**c**) Fractional variance (black bar) and cumulative fractional variance (black line) for the first 50 PC modes from an EDA of the Grand ensembles of MD configurations. The arrow points to the mode index in which the cumulative fractional variance is 0.8. (**d**) Projections of 30,000 conformations of the Grand ensemble, colored according to the original trajectories as in (a), onto the principal component PC_G_(1) and PC_G_(2) calculated by EDA.

The conformational flexibility of the apo-protein in solution was addressed by molecular dynamics (MD) simulations from the X-ray (5DAZ) apo-protein as indicated in the *Materials & Methods*. To evaluate the dynamic behavior of apo-Scabin in solution three 105 ns runs were performed at room temperature (*T* = 22 °C) and named “Run_a_”, “Run_b_”, and “Run_c_”, respectively. Fig 2a shows the RMSD time-course of 100 ns for the production phase of each trajectory after an optimal iterative superposition of 10,000 conformations of each ensemble (“Ens_a_”, “Ens_b_”, and “Ens_c_”, respectively) based on the C_α_-trace of *N* = 160 atoms of the segment Ala41–His200 (the entire protein, except the five residues, Lys36–Pro40, due to their high mobility). All the conformations sampled within the 20–75 ns time interval showed a high structural similarity in the respective average conformation (“Trace_a_”, “Trace_b_”, and “Trace_c_”, respectively) of each ensemble. After t ~75 ns, each trajectory deviates from its average conformation, Run_a_ and Run_b_ followed a variable, while Run_c_ showed an attenuated (i.e., a more confined trajectory) RMSD_α_ time-course.

After the three average traces were superposed onto the X-ray structure (5DAZ), the average pairwise RMSD_α_ was 1.14 Å. This relatively low value suggests that the overall domain folded structure is preserved in each of the average coordinates for the three simulations. Hence, the fluctuations recorded in each ensemble may reflect part or all the equilibrium dynamics of Scabin in solution. Trace_a_ deviates the most with respect to the X-ray conformation (RMSD_α_ = 1.18 Å), particularly in the region corresponding to the exposed ARTT-loop, PN-loop, and β_6/7_-turn (not shown).

An essential dynamic analysis (EDA)[22] was performed for each conformational ensemble. The calculation of the cumulative fractional variance as a function of the principal component (PC) index (PC(*i*), *i* = 1… *N*-6 modes) shows that the first 20 (out of 474) PC modes for the ensembles, Ens_a_ and Ens_b_, and the first 30 modes for the ensemble Ens_c_, account for ~80% of the total variance in the atomic fluctuations (Fig 2b). However, the low pairwise cosine-overlap (the highest absolute cosine-overlap is 0.64 between PC_a_ (3) and PC_c_ (4) modes) of the individual dominant PC modes among the three trajectories reflects the specific nature of the fluctuations for each trajectory.

Based on the previous results, a “Grand” ensemble (“Ens_G_”) of 30,000 conformations was made by combining the three MD trajectories, and the Ens_G_ was interposed to obtain the average C_α_-trace (“Trace_G_”). Trace_G_ deviates 0.79 Å from the X-ray structure and only 0.56 Å from Trace_c_. An EDA performed over Ens_G_ showed that the combined effect of the 15 most dominant PC_G_ modes of fluctuations (PC_G_(*i*), *i* ≤ 15) describe the “*essential dynamics”* (set arbitrarily at 80% of the total variance) of apo-Scabin at room temperature (Fig 2c).

**Fig 3:**
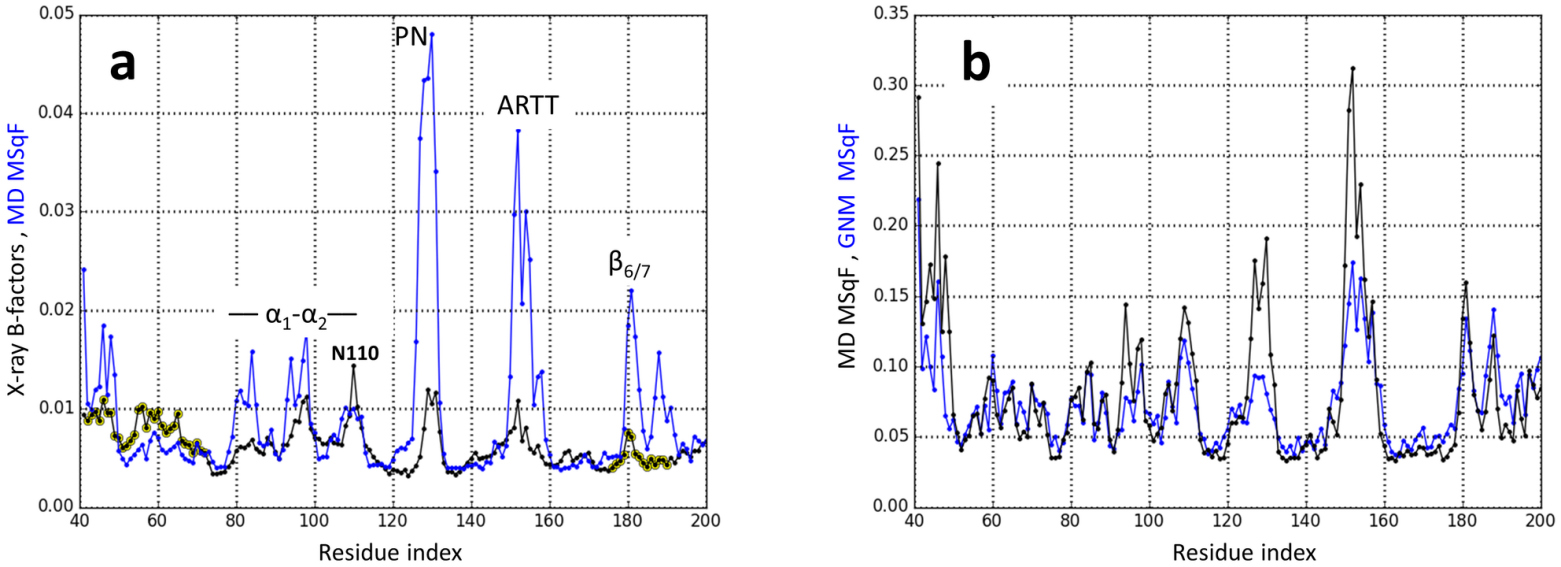
Experimental and simulated mobility profiles of apo-Scabin. (**a**) Normalized MSqF from the Grand ensemble of conformations (blue) and normalized *B*-factors for the 5DAZ structure (black). Highlighted with yellow are the crystallographic *B*-factors of residues belonging to SS_1_ and SS2 loops. (**b**) Normalized MSqF from the Grand ensemble of conformations by only using the set [5,159] of PC_G_ modes (black), and normalized MSqF from a GNM on the Trace_G_ conformation by using all, [1,159], GNM_G_ modes (blue). For the GNM, the (force constant took the value γ(*N*_1_,*N*_2_) = 1 for any *N*_1_ and *N*_2_ node of the network, except when one or both nodes belong to specific segments or residues (*e*.*g*., PN-loop, SS_1_-loop, Cys42, etc.) as follows: γ(PN,*N*_2_) = γ(ARTT,*N*_2_) = 0.64, γ(SS_1_,SS_1_) = γ(SS_2_,SS_2_) = 1.07, and γ(C42,C72) = γ(C176,C190) = 3.57.

Fig 2d shows the projection of Ens_G_ conformations onto the plane formed by the two lowest (highest variance) PC_G_ indexes: PC_G_(1) and PC_G_(2). The combined trajectories present a clear anharmonicity for fluctuations along the PC_G_(1) mode (S1a Fig). The different trajectories cover sub-spaces and span up to 40 Å of total fluctuation (*e*.*g*., between conformations of the Run_a_ and Run_b_ trajectories). Likewise, the fluctuations described by PC_G_(2) are also anharmonic, as can be seen by the presence of two clusters of Run_a_ conformations with a total variation of up to ~30 Å (S1b Fig). Fig 3a shows the normalized profile of C_α_ mean square fluctuations (MSqF) derived from the Grand ensemble. Most of the MD structural variations correspond to fluctuations in the exposed PN and ARTT loops, in the α_1_-α_2_ motif, and in the turn-turn segment of the SS_2_-loop.

### Assessment of the MD dynamics of apo-Scabin

In crystallography, temperature factors (or *B*-factors) quantify the uncertainty of each atom by describing its mean square displacement in the context of the protein crystal. In this sense, it is pertinent to compare the MD MSqF profile obtained from the Grand ensemble with the *B*-factors profile of the apo-Scabin X-ray structure (5DAZ). Fig 3 depicts the normalized profiles for both the MD MSqF values versus the X-ray B-factors, which show a modest agreement with a Pearson correlation coefficient of *r* = 0.50. However, the contrast in the mobilities between the different elements and segments (*e*.*g*. ARTT-loop *v*. α_1_) is more evident for the fluctuations of the toxin in solution than for the thermal fluctuations estimated from the crystallized structure.

An inspection of the crystal unit parameters for the apo-Scabin structure shows close (< 4.5 Å) intermolecular contacts throughout the whole molecule (S2 Fig). It is expected that differences (beyond a scaling factor) will be evident for the experimental fluctuations in solid-phase (crystal structure) and the MD fluctuations in solution. For example, the mobility of α_2_ (S105–V109) is controlled by interactions with a neighboring molecule in the crystal. Accordingly, Asn110 is free of crystal interactions and reports a relatively high local mobility in agreement with its *B*-factor (Fig 3a). Likewise, most of the SS_1_-loop is free of the effect of crystal packing, hence its relatively high mobility according to the *B*-factors. The case of the SS_2_-loop is the opposite–the mobility of the loop is constrained by crystal contacts (S2 Fig). In fact, if the SS_1_ and SS_2_ loops are not considered, the correlation between *B*-factors and MD MSqF profiles for the sequence range (G73–V165) increases to *r* = 0.60. In summary, the restricted mobility for the crystallized Scabin dictates that the *B*-factors do not provide a good indication of the dynamics of various structural elements as it pertains to its equilibrium dynamics.

To further assess Scabin molecular dynamics as described by MD simulations, a Gaussian network modeling (GNM) approach was implemented. For this approach, a network model that connects the C_α_-atoms (nodes) with a homogenous force constant was defined, and the cutoff that determines the Kirchhoff matrix (the connectivity of the system) was optimized to maximize the Pearson coefficient between the MD MSqF and the GNM MSqF. Likewise, the number of modes to include in the MD MSqF calculations was a variable to maximize the correlations. The rationale is based on the observation that the main PC modes (lowest-indexed modes) usually present a high anharmonic character (S1 Fig); consequently, the most dominant PC modes would not overlap with the harmonic modes calculated by the elastic approach. Table 1 reveals the correlation coefficient between paired MSqF profiles by omitting the PC(1) and PC(2) modes for the individual ensembles, and the PC_G_(1)–PC_G_(4) modes for the Grand ensemble.

**Table 1:** Correlation between simulated and calculated mobilities in Scabin.

Trace	PC modes	Correlation coefficient (r)^a^
Trace_a_	3–159	0.85
Trace_b_	3–159	0.76
Trace_c_	3–159	0.73
Trace_G_	5–159	0.78

^a^Pearson correlation coefficients between the MD MSqF of each independent (a—c) or Grand (G) trajectories and the GNM MSqF from the average coordinates. The number of PC modes included in the calculations is also specified.

Furthermore, considering the additional connectivity that arises due to the two disulfide bonds and the differences in packing density of the ARTT and PN loops, a custom-made force-function was defined to reproduce the MD MSqF profile. With these residue-dependent force constants and a cutoff of 12 Å, the correlation coefficient for the Grand ensemble was maximized to *r* = 0.85 (Fig 3b). The strong correlation between the MSqF derived from the combined 155 highest-ranked (index: 5–159, in 3*D*-modes) PC modes of fluctuations and all (index: 1–159) of the GNM modes of fluctuations, makes both methodologies self-consistent in terms of describing the harmonic dynamics of Scabin in solution.

#### Hydrogen-deuterium exchange for apo-Scabin

**Fig 4:**
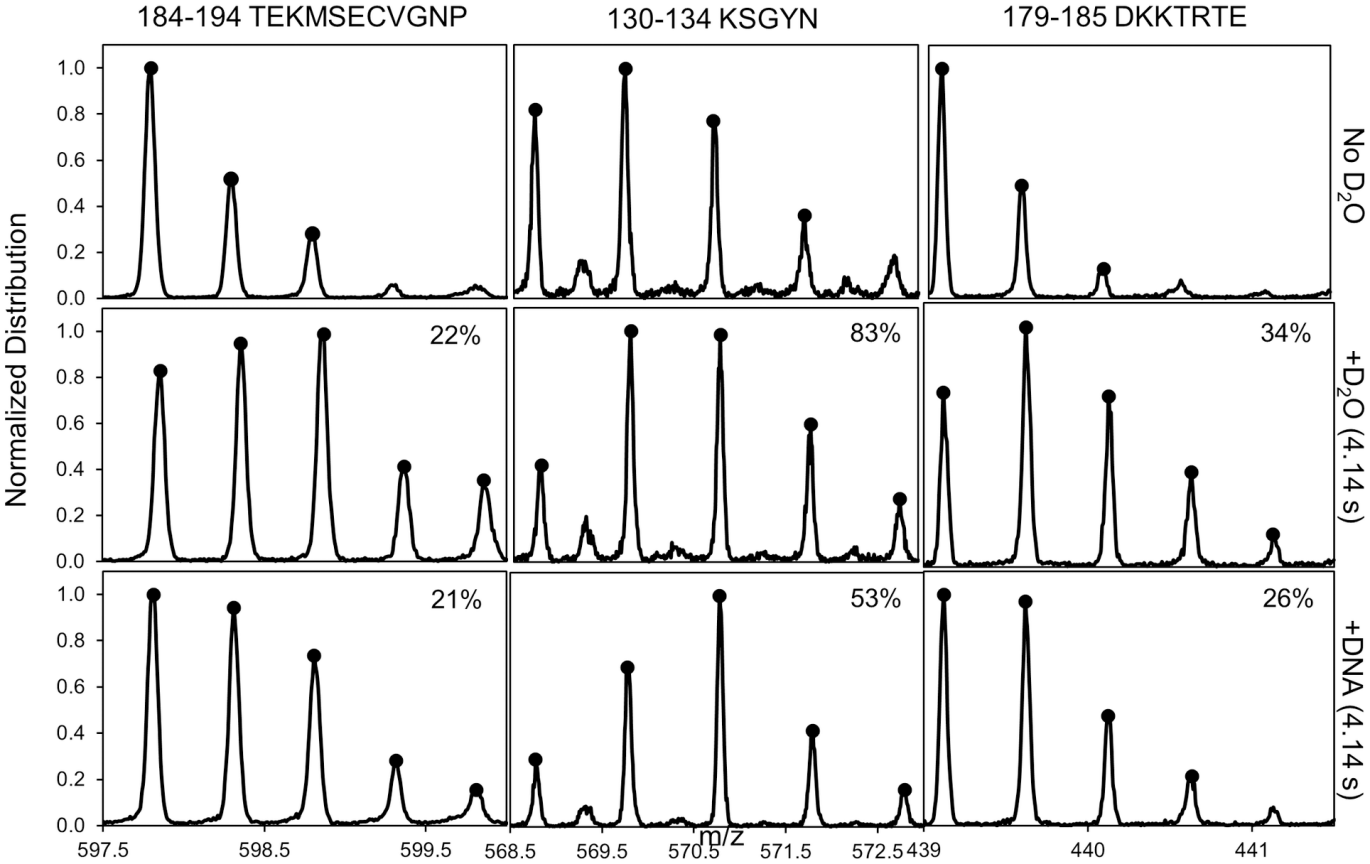
HDX mass spectra for pepsin digestion of the Scabin-DNA complex. Representative spectra of peptides obtained from pepsin digestion of non-deuterated apo-Scabin (top), labeled with a 4.1 s HDX pulse (middle) and complexed with DNA (bottom) labeled by a 4.1 s HDX pulse. Representative peptides are depicted that showed no change (left) or a decrease in uptake located in the PN-loop (middle) and β_6/7_-turn (right). Percent deuterium uptake is indicated on each spectrum.

Hydrogen-deuterium exchange coupled with mass spectrometry (HDX-MS) was performed on apo-Scabin to quantify the exposure/flexibility of Scabin structural elements in solution. With a total sequence coverage of 98%, the isotopic distribution for each peptide of length *L* was measured at a single-reaction time-point, and the fractional deuteration level, $0\leq {\overset{-}{D}}_{v}\leq 1$, of each peptide was calculated considering a binomial distribution of deuterium uptake as described in the *Materials & Methods*. However, the assignment accuracy of the average deuteration content at the peptide-level to the individual constituting residues decreases with the length and structural variability of the peptides. In fact, all longer peptides (*L* > 12) gave ${\overset{-}{D}}_{v}$ values lower than any of the corresponding overlapping (either completely or partially) peptides. In this sense, only 53 peptides with lengths between 3 and 12 residues were considered for the analysis. HDX experimental data and analysis for some representative peptides from pepsin digestion of apo-Scabin are shown in Fig 4. A color-coded representation of the fractional deuterium uptake for Scabin peptides and the average values at the residue-level, ${\overset{-}{D}}_{i}$, are shown in S3 Fig.

Nevertheless, to overcome this limitation and to estimate “missing” ${\overset{-}{D}}_{i}$ values, an *in-silico* approach to calculate the “limiting” probability of H/D exchange at the residue-level was implemented as described in the Materials & Methods. Thus, the MSqF_*i*_ ($=\langle {\Delta R}_{i}^{2}\rangle$) derived from the Grand ensemble of MD conformations, instead of the MSqF_*i*_ = [Γ^−1^]_*ii*_) from the GNM calculations as defined in Eq 8, was used to estimate the theoretical energy profile of H/D exchange according to

$${\Delta G}_{i,HDX}\propto \frac{{\Delta G}_{i,MD}}{{\Delta R}_{i}^{2}}\propto \frac{1}{\langle {\Delta R}_{i}^{2}\rangle }$$ (1)

**Fig 5:**
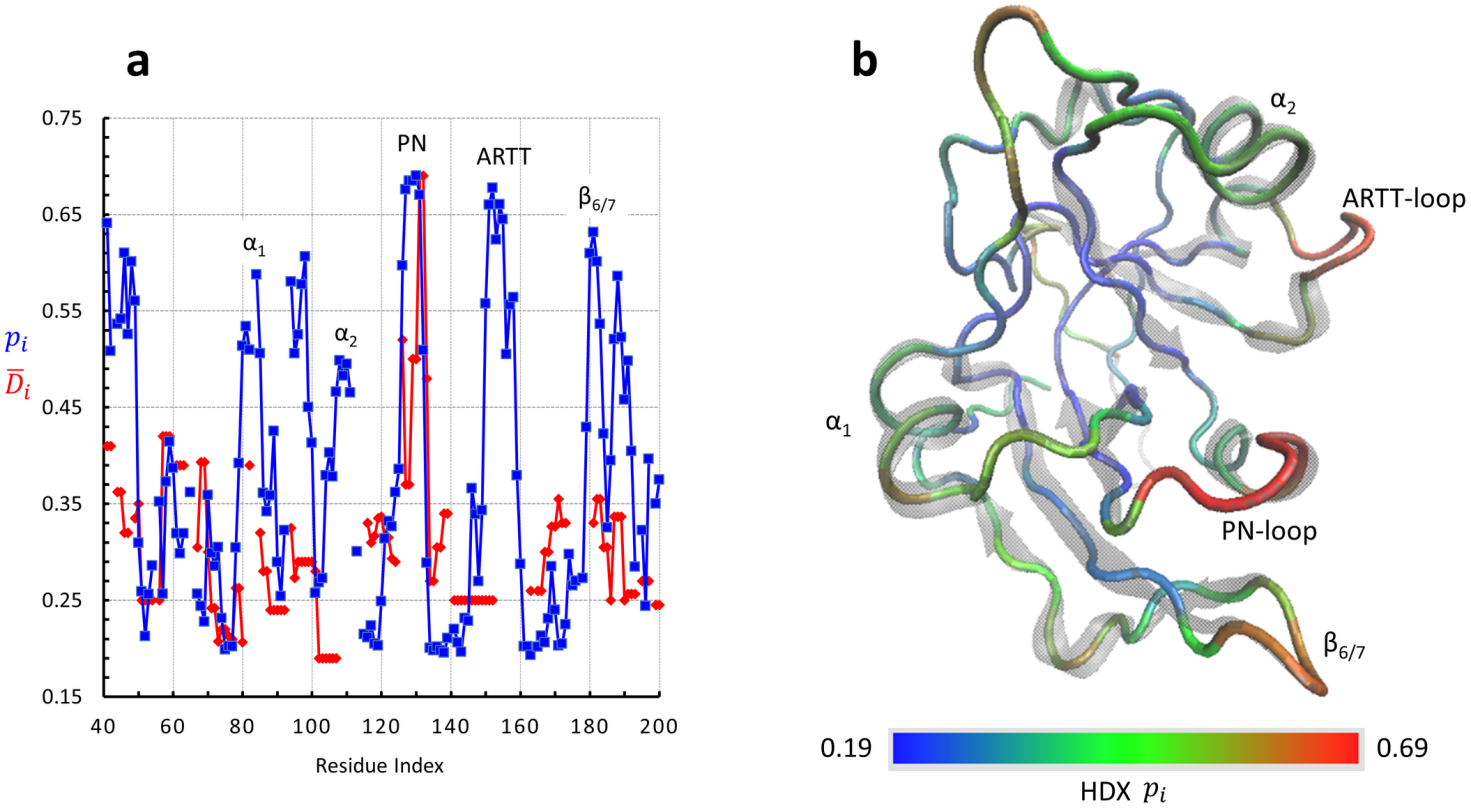
Mapping of H/D exchange for apo-Scabin. (**a**) Experimental average deuteration level based on the average of 53 peptides with *L* ≤ 12 residues (red), and theoretical relative probability of deuteration based on the MD MSqF and scaled to cover the experimental range (blue) for each backbone N-amide of apo-Scabin. (**b**) Tube representation of the Trace_G_ apo-Scabin spectrally colored from blue (*p*_i_ = 0.19) to red (*p*_i_ = 0.69) according to the relative H/D exchange probability plotted in (a). Rendered by VMD.

Substituting Δ*G*_*i*,*HDX*_ from Eq 11 into Eqs 9 and 10 and solving for *p*_*i*_, the calculated profile of relative probabilities of deuterium uptake of the apo-Scabin backbone N-amides, scaled to the experimental 0.19–0.69 range, is shown in Fig 5a. A spectrally-colored representation of the *p*_*i*_ mapped onto the Trace_G_ is shown in Fig 5b.

Overall, the calculated H/D exchange probabilities (*p*_*i*_) agree with the experimental values at the residue-level (${\overset{-}{D}}_{i}$) in the sense that peaks of high probabilities are in exposed loops and coiled structures, while more ordered secondary-structure elements are valleys of low propensity for exchange. Major deviations come from constant experimental values for some segments estimated by a unique peptide (*e*.*g*. α_2_ helix), and in some cases, the peptide encompassing a variety of secondary-structure elements (*e*.*g*. peptide G142–H153) (S3 Fig). Nevertheless, the experimental-theoretical disagreement may have its origin in: (*i*) implicit differences when a temporary H/D exchange is compared with an equilibrium value, (*ii*) inherent limitation at the peptide-level calculation by using a binomial distribution when there are residues with highly contrasting H/D exchange probabilities, and (*iii*) the effective propensity for the H/D exchange of a backbone N-amide might not uniquely be determined by the energy of the close ↔ open transition based on the atomic fluctuations as stated by Eq 7 (*structural* effect), but also may be controlled by the average shielding (*steric* effect) and an inductive influence (*specific* effect) produced by the side-chains of the residues in the vicinity of the amide site.

#### Collective and essential fluctuation modes

**Fig 6:**
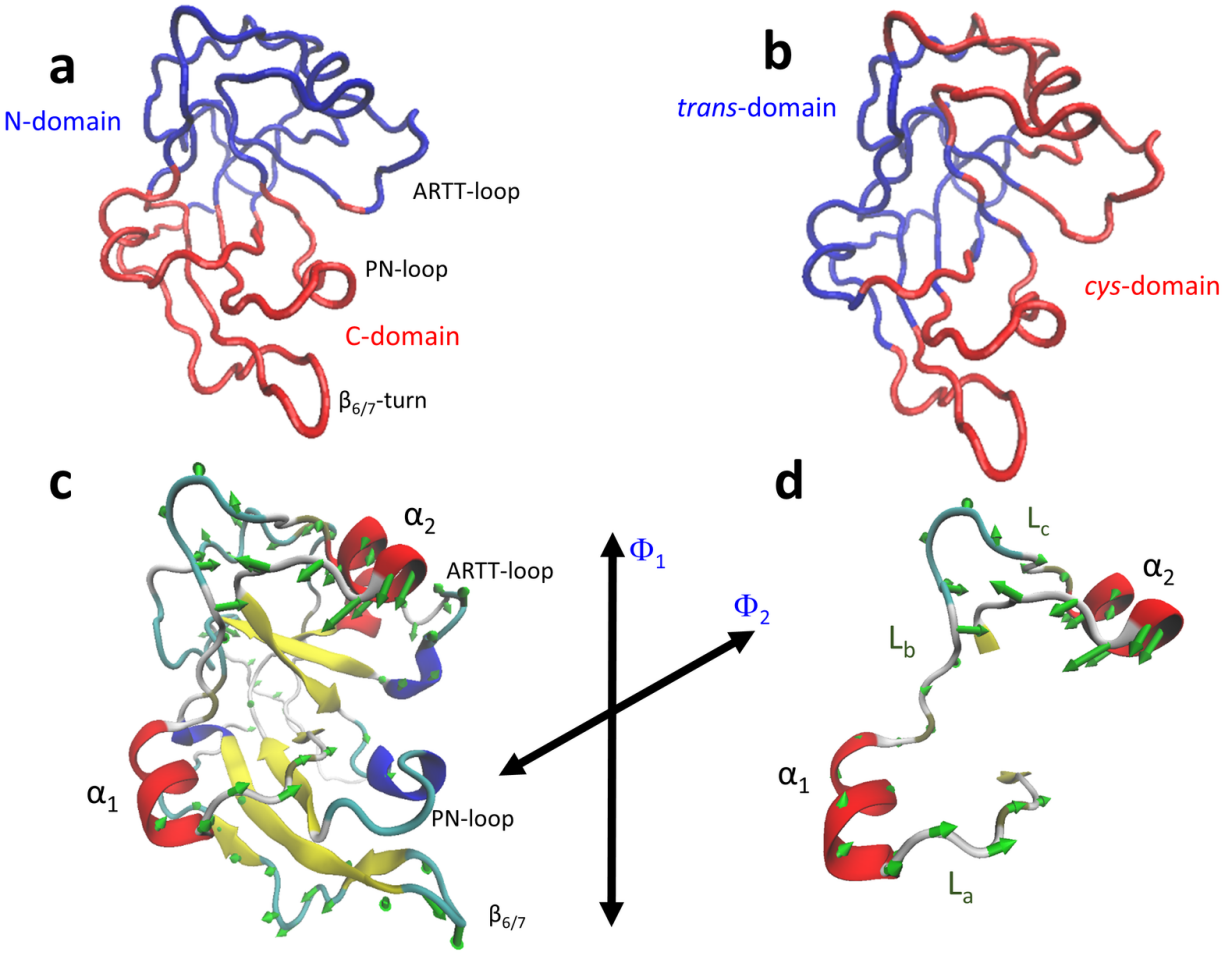
Domains of motions for the slowest GNM modes of apo-Scabin. Tube representation of the Trace_G_ apo-Scabin colored according by blocks of correlated movements as defined by the (**a**) GNM(1) and (**b**) GNM(2) modes of fluctuation. Rendered by NMWiz in VMD. *Deformation into the essential space of apo-Scabin*. Depiction of vectors resulting from combining the PC_G_(1)–PC_G_(15) principal component modes that characterize the direction and relative magnitudes of the deformations that describe the *essential dynamics* of apo-Scabin, (**c**) Deformation vectors for all the C_α_-atoms except for the PN loop, scaled for an <RMSF> of 1.21 Å and only those vectors are shown whose RMSF ≥ 0.5 Å. Φ_1_ and Φ_2_ are defined as two arbitrary axes of displacement. (**d**) Deformation vectors for all the Cα-atoms of the α_1_-α_2_ motif.

The GNM modes calculated over Trace_G_ can be useful to visualize the coupling/uncoupling in the main (lowest-indexed) harmonic fluctuations between the different structural elements. In this sense, the most collective mode of movement, elicited by GNM(1), shows two spatial regions with an anticorrelated movement with respect to each other. These regions correspond to tertiary-structure elements that might be called a *N-domain* (formed by the β_I_ sheet, PN-loop, α_1_, and the β_6/7_-turn, among other segments) and a *C-domain* (formed by the β_II_ sheet, ARTT-loop, and α_2_, among other segments), respectively (Fig 6a). The second most collective mode of fluctuation, elicited by GNM(2), describes two topological regions with a correlated movement that might be called a *cis-domain* (formed mainly by the PN and ARTT loops, α_2_, and the β_6/7_-turn) and a *trans-domain* (formed by α_1_ and other elements), according to whether the secondary-structure element is facing the NAD^+^-binding cavity, *cis* side (Fig 6b).

**Fig 7:**
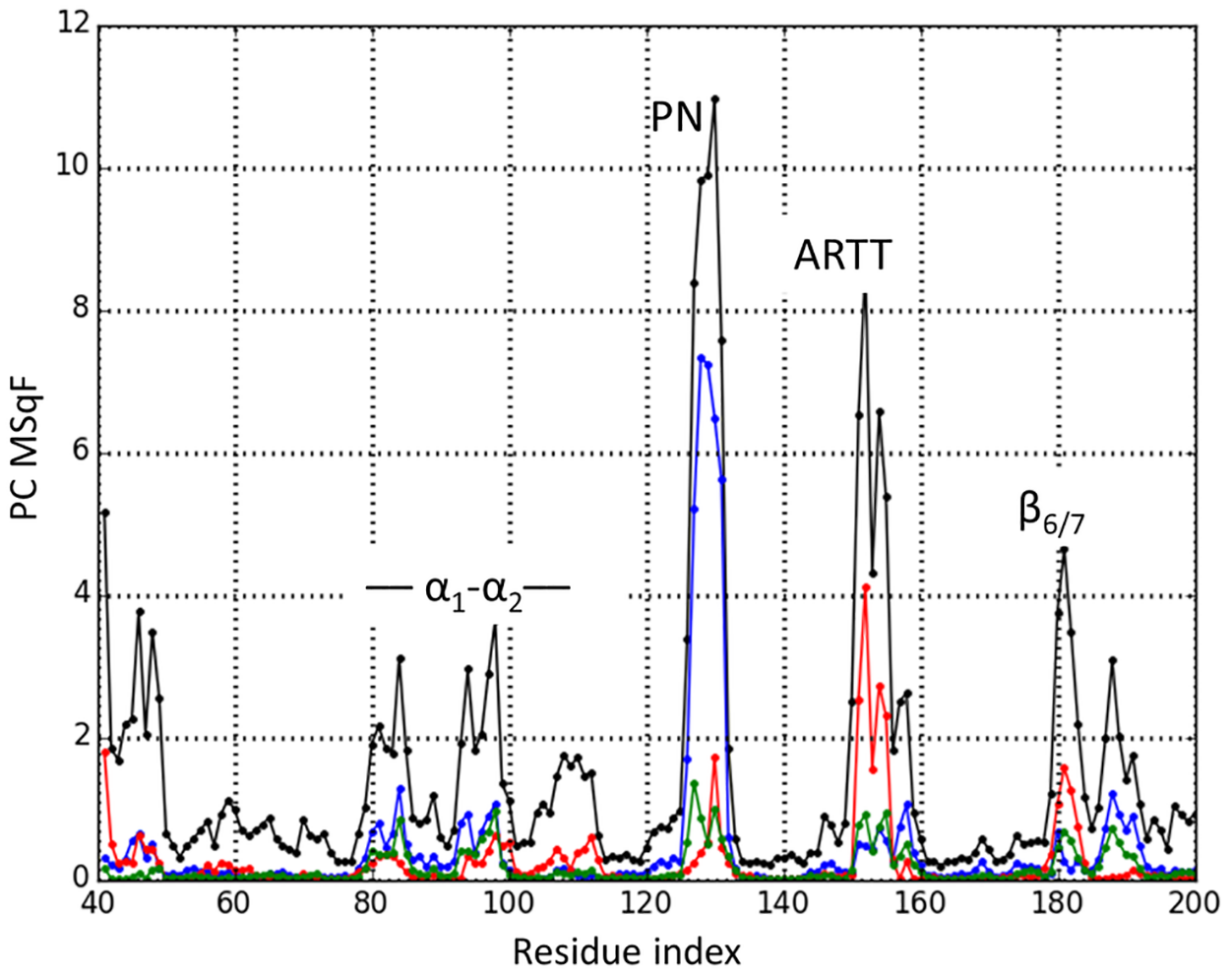
Dissection of apo-Scabin mobility. Total sum of square fluctuations (black) of the Grand ensemble conformation, and the contribution of the lowest-indexed PC modes. SSqF from PC_G_(1) in blue, PC_G_(2) in red, and PC_G_(3) in green.

However, only by using the PC modes can the *3D*-directionality of the deformations elicited by harmonic and anharmonic components be visualized. After omission of the ARTT and PN loops due to their high mobilities, the vectors that determine the combined fluctuations elicited by the first 15 PC modes (the *essential space*) can be depicted in Fig 6c. In this combined mode of fluctuation, the PN-loop (vectors not shown) fluctuates in anticorrelation following the Φ_1_ direction with both the β_6/7_-turn and α_2_, while the two latter elements move in correlation with respect to each other. As a result, the α2 –PN distance decreases when the β6/7 –PN distance increases, and *vice versa*. The ARTT-loop and α_1_ fluctuate in anticorrelation by following the Φ_2_-direction (quasi perpendicular to Φ_1_). Regarding the fluctuations of the α_1_-α_2_ motif, it is dominated by fluctuations of α_2_ (Fig 6d). Upon dissecting the MD MSqF per individual PC mode (Fig 7), the PC_G_(1) fluctuation mode is largely determined by changes in the conformation of the PN-loop; in contrast, PC_G_(2) is determined mainly by changes in the conformation of the ARTT-loop (primary) and in the β_6/7_-turn (secondary). The contribution of the PC_G_(3) is more equally distributed among all these elements, although it is considerably lower in amplitude.

### II. The Scabin·NAD^+^·DNA complex

**Fig 8:**
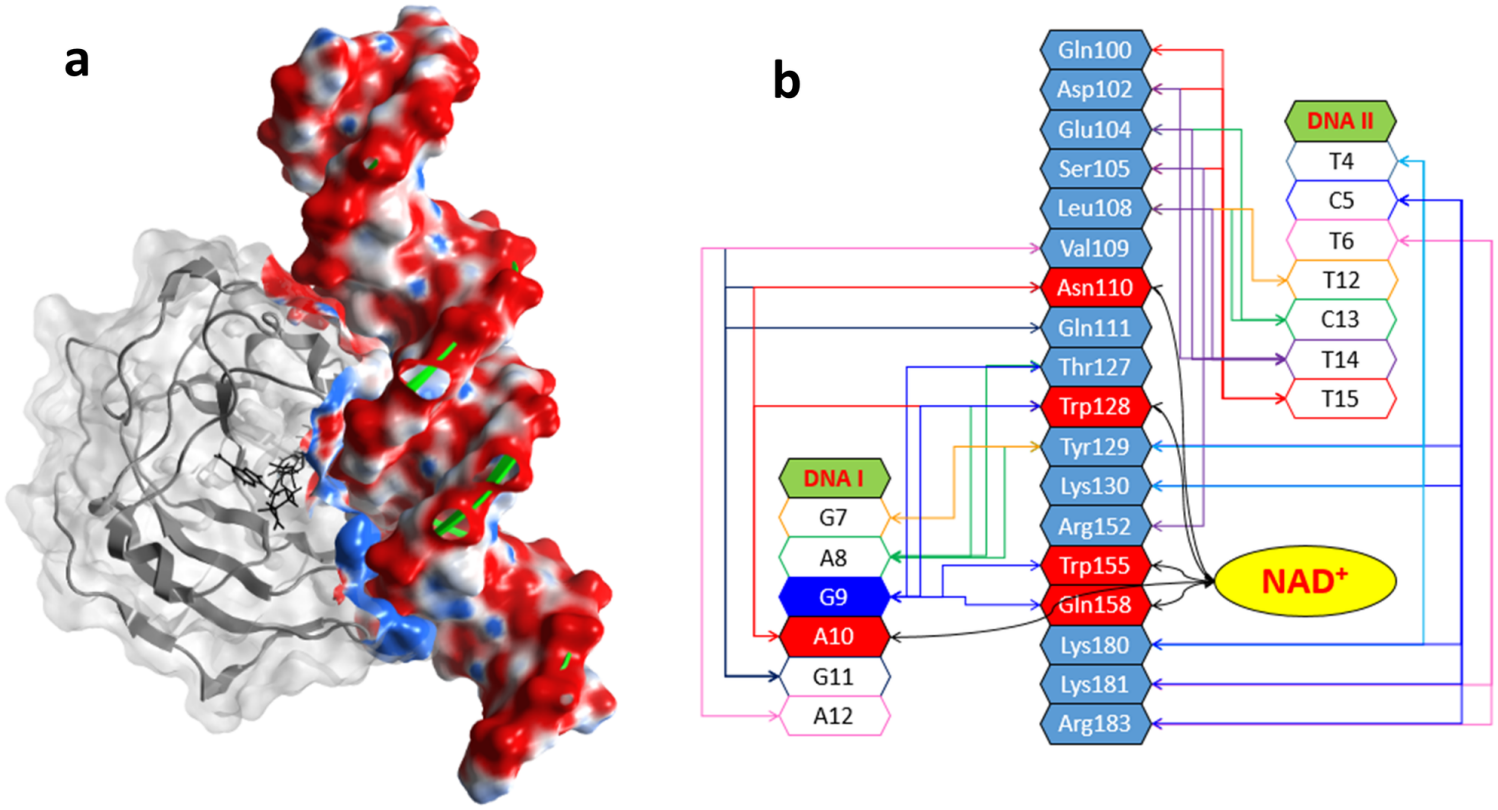
(**a**) *The Scabin·NAD*^+^*·DNA complex model*. Depiction of the molecular surface of the ternary Scabin·NAD^+^·DNA model. Scabin in gray ribbons and with light gray translucent surface except for the contact interface, which is colored according to the electrostatic potential; NAD^+^ is shown in black sticks, and the dsDNA molecule in green ribbons and molecular surface colored according to the electrostatic potential. (**b**) *Scabin·NAD*^+^*·DNA interactions*. A schematic representation of the network of interactions between 18 Scabin residues (in light blue) and 13 bases from both DNA strands. Also, the interactions between NAD^+^ and 4 common residues (in red); the DNA1 base is shown in red. A centrally located guanine base is shown in dark blue at the 9^th^ position of the DNA_I_ strand and is a potential candidate as the nucleophile for the ADP-ribosylation reaction.

An *in-silico* model of Scabin complexed with NAD^+^ and with a 21-bp DNA oligomer was generated by a docking protocol as outlined in the *Materials and Methods*. In the Scabin·NAD^+^·DNA ternary complex (Fig 8a), the active conformation of Scabin reveals a smaller structural difference between the apo-Scabin (5DAZ) structure and the Scabin·NADH complex (5TLB), with RMSDα values of 0.88 Å and 0.95 Å, respectively. Regarding the active conformation of the NAD^+^, its binding pose is essentially identical to that of NADH in the Scabin·NADH complex (5TLB) and to that of NAD^+^ in the Pierisin-1·NAD^+^ complex (5H6J), except for the dihedral P_N_-O–C-C [17].

**Fig 9:**
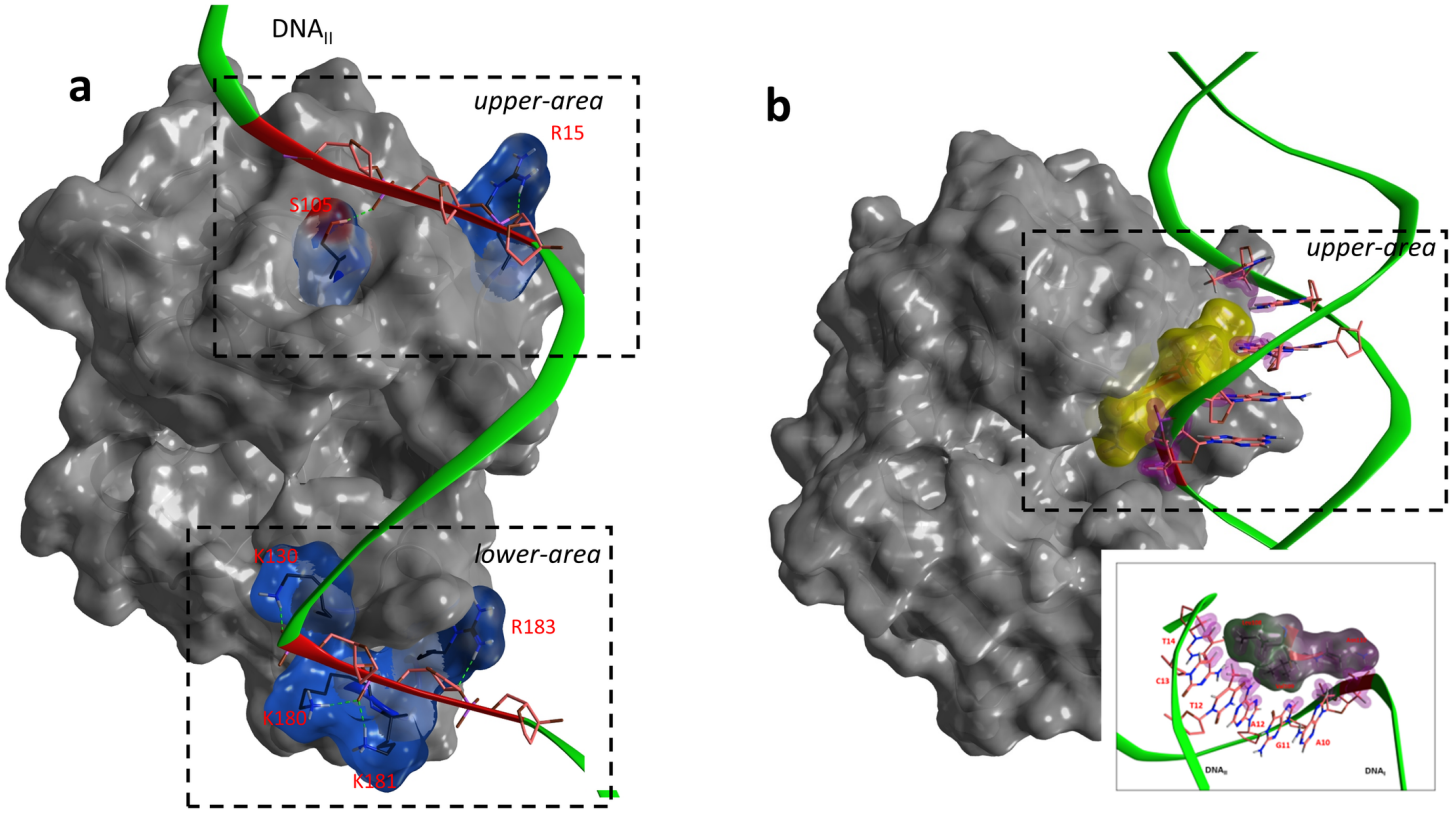
(**a**) *Upper and lower Scabin·DNA electrostatic contact areas*. Depiction of the H-bonds and electrostatic components of interaction between Scabin and the DNA_II_ strand at two distant contact points. Scabin is represented by an opaque, dark gray molecular surface, but with a translucent surface colored according to the electrostatic potential at the contact residues. The backbone of DNA_II_ in green ribbons but colored in red at the contact bases. (**b**) *Hydrophobic interactions in the Scabin·DNA upper contact area*. Depiction of exposed hydrophobic residues embedded in α_2_ within a major DNA groove. Scabin is represented by an opaque, dark gray molecular surface, but with slightly translucent, amber surface for the hydrophobic motif. *Inset*. Details of participating residues (in gray surface) and bases. In both, the backbone of DNA in green ribbons, and stick depiction of 5 interacting bases with C-atoms in orange and purple shells highlighting the van der Waals surface of contacting atoms.

**Fig 10:**
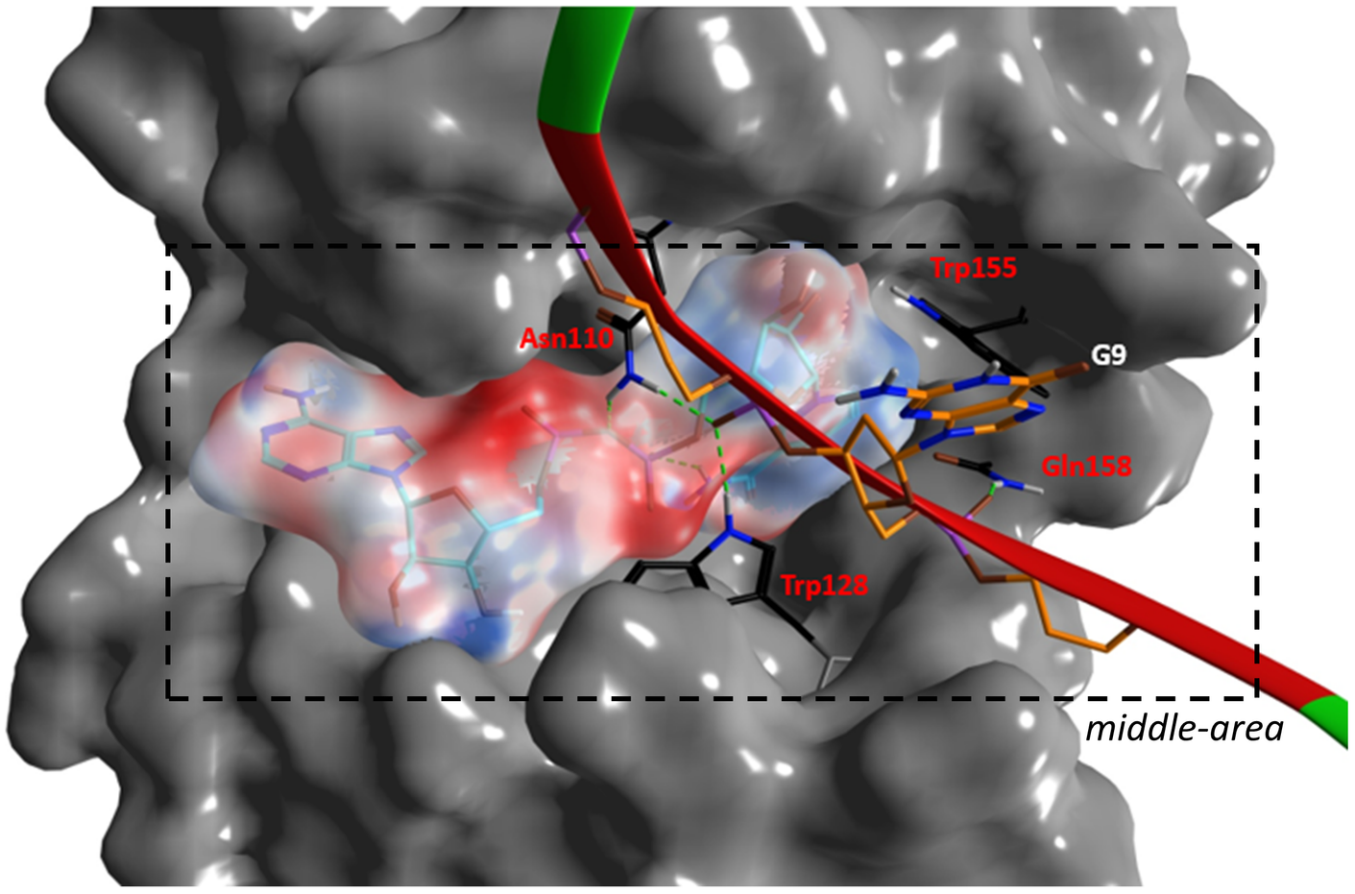
Scabin·NAD^+^·DNA interactions. Depiction of the interacting residues of Scabin in the *middle-area* with DNA_I_ strand and NAD^+^ molecule. Scabin is represented by an opaque, dark gray molecular surface, but with an all-atoms depiction of 4 bridge residues in black C-atoms. The NAD^+^ molecule is shown in stick cyan C-atoms with a translucent molecular surface colored according to the electrostatic potential. The backbone of DNA_II_ is shown in green ribbons but colored in red at the contacting bases. All-atoms of a guanine base in the 9^th^ position are depicted.

**Fig 11:**
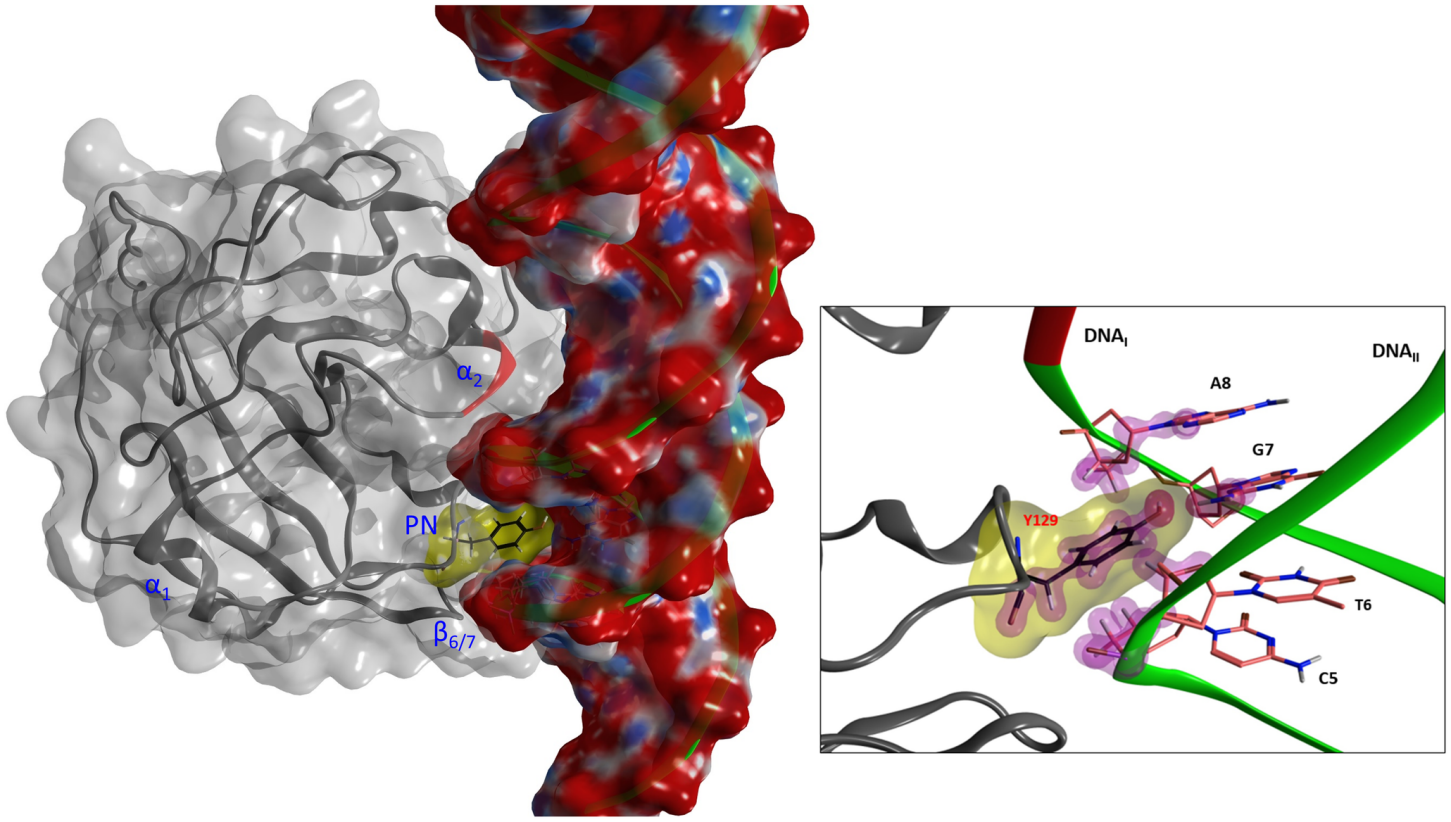
Interacting motifs of the Scabin· DNA complex. The DNA-contact motifs of Scabin are shown including α_2_, the PN-loop and the β_6/7_-turn. Scabin is represented by dark gray ribbons and with a translucent, light gray molecular surface. Also, an all-atom depiction of Tyr129 with yellow molecular surface embedded into a minor DNA-groove. The dsDNA molecular surface is colored according to the electrostatic potential. *Inset*. Details of Tyr129 interactions into the minor DNA groove. Purple shells highlight the van der Waals surface of contacting atoms.

According to our model, dsDNA binds to Scabin with a large contact surface that encompasses 18 residues and 13 bases on both DNA strands (Fig 8b). This large contact surface can be topologically subdivided according to the location of the contacting residues in (*i*) a *lower-area* which mainly binds the outermost DNA_II_ strand (Fig 9), (*ii*) a *central-area* which mainly binds the innermost DNA_I_ strand (Fig 10), and (*iii*) *upper-area* which binds both DNA strands (Fig 11).

The *lower-area* of contact is mainly characterized by electrostatic interactions between positively charged residues and the negatively charged DNA backbone (Fig 11a). Indeed, the DNA_II_ strand is tethered with strong salt-bridges by side-chains of the cluster 180-KKxR (β_6/7_) and with Lys130 (PN-loop) (basic residues). In the *upper-area*, the DNA_II_ strand is also held by a strong salt-bridge with Arg152 (ARTT-loop) and by an H-bond with Ser105 (α_2_). However, hydrophobic interactions between α_2_ residues and bases in the DNA major groove better characterize the contact in this area. In effect, the side-chains of Leu108 and Val109 are surrounded by the substituents of 5 bases from both DNA strands (Fig 11b).

In the *middle-area* of the Scabin protein, four residues, Asn110, Trp128, Trp155, and Gln158, contact both the DNA_I_ strand and the NAD^+^ molecule (Fig 10). Trp155 (ARTT-loop) makes van der Waals interactions with the side-chain of the 9^th^ base of DNA_I_, which happens to be a guanine base (see later). Asn110 (L_c_), Trp128 (PN loop), and Gln158 (ARTT loop) contact the DNA_I_ backbone by van der Waals and H-bonds interactions. In addition, Thr127 (α3) forms a contact with the DNA_I_ backbone (not shown). However, it is Tyr129 (PN loop) that is the key residue for controlling the interaction in this area. Specifically, Tyr129 intercalates into a minor dsDNA groove and contacts both DNA strands by interacting with the backbone of 4 DNA bases and with the side-chain of the 7^th^ base of DNA_II_ (Fig 11b).

#### Binding properties of Asn110 in Scabin

The kinetics and binding properties of three residues (Trp128, Trp155, and Gln158) that bridge the NAD^+^ and the dsDNA substrates were previously reported where site-directed mutants of Scabin were prepared and the variants were studied and compared to the WT toxin [14]. Herein, the fourth-member of this proposed Scabin·NAD^+^·DNA bridge, Asn110, was investigated with the N110A Scabin variant. Regarding the NAD^+^ substrate, the affinity was evaluated by measuring the *K*_M_ of GH activity using the εNAD^+^ analog [9, 14]. In Scabin, the Asn to Ala does not impair the enzyme’s interaction with the di-nucleotide substrate, since the Michaelis-Menten constant of the N110A variant, *K*_M_(εNAD^+^)_N110A_ = 66 ± 12 μM, is nearly identical to the WT value–*K*_M_(εNAD^+^)_WT_ = 68 ± 3 μM. Regarding the dsDNA substrate, the affinity was evaluated by measuring the binding constant of a cyanine-3 tagged dsDNA oligonucleotide (21 base pair; see Material & methods). In Scabin, the Asn to Ala substitution impairs the interaction with dsDNA, as the dissociation constant of the N110A variant, *K*_D_(21bp-DNA)_N110A_ = 82 ± 5 μM, is slighter higher (and statistically significant) than the dissociation constant of the WT toxin of *K*_D_(21bp-DNA)_N110A_ = 51 ± 4 μM.

#### Dynamics of the Scabin·DNA complex

HDX experiments were performed with Scabin in the presence of dsDNA, as indicated in *Materials and Methods*. The average deuteration content at the residue-level, ${\overset{-}{D}}_{bound}$, was calculated based on the peptide-level fractional deuteration for those with *L* ≤ 12 residues in length (S4 Fig), and the difference in the deuteration, $\Delta \bar{D}(={\bar{D}}_{bound}-{\bar{D}}_{apo})$ for those residues with experimental values for apo and the dsDNA-bound forms of Scabin were calculated (Table 2). The negative variation for most of the reported peptides may be explained by a restriction in the mobility and/or a steric shielding associated with DNA binding. This is clearly the case for the PN loop (or the whole β_2_–PN–β_3_ super-structure) and the β_6/7_-turn. Surprisingly, the Gly141–Ile149 segment showed a positive variation. Increasing the dynamics of segments allosterically linked to a binding motif (an increase of HDX upon ligand binding) has been reported previously [23]. This Gly 141 –Ile149 segment includes the β_4_-α_4_ super-structure at the *trans* side of the ARTT `loop; the increased exposure/mobility might be the result of a “hinge” deformation caused by the anchoring of ARTT *cis* residues (*e*.*g*. Arg152, Trp155) when interacting with the DNA molecule.

**Table 2:** Variations in the deuteration level of Scabin due to the DNA binding.

Element	Res	*D*_bound_	D*D*_bnd-apo_	Element	Res	*D*_bound_	D*D*_bnd-apo_	Element	Res	*D*_bound_	D*D*_bnd-apo_
**SS**_**1**_**-loop**	Val57	0.34	-0.08	**PN loop**	Tyr120	0.28	-0.05		Thr148	0.40	0.15
	Asp58	0.34	-0.08		Asp123	0.29	0.00		Ile149	0.40	0.15
	Val59	0.34	-0.08		Leu124	0.29	0.00	**β**_**6/7**_**-turn**	Lys181	0.27	-0.06
	Arg61	0.37	-0.03		Lys130	0.44	-0.06		Thr182	0.27	-0.09
	Ile62	0.37	-0.03		Ser131	0.44	**-0.25**		Arg183	0.27	-0.09
	Thr63	0.37	-0.03		Gly132	0.44	**-0.25**	**β**_**7**_	Thr184	0.26	-0.05
	Thr71	0.21	-0.03	**β**_**3**_	Tyr133	0.44	-0.04		Glu185	0.26	-0.05
	Cys72	0.21	-0.03		Asn134	0.33	0.06		Met186	0.21	-0.04
	Gly73	0.21	0.00		Tyr135	0.22	-0.05		Ser187	0.21	-0.13
	Thr74	0.21	-0.01		Tyr136	0.22	-0.08		Glu188	0.21	-0.13
	Leu75	0.21	-0.01		Ile137	0.22	-0.08		Glu189	0.21	-0.13
	Tyr76	0.21	0.00		Asp138	0.22	-0.12		Cys190	0.21	-0.04
	Arg77	0.21	0.00		Gly141	0.48	0.23	**β**_**8**_	Val191	0.21	-0.05
**β**_**2**_	Val116	0.29	-0.04		Gly142	0.48	0.23		Gly192	0.21	-0.05
	Ser117	0.28	-0.03	**β**_**4**_	Val143	0.48	0.23		Asn193	0.21	-0.05
	Thr118	0.28	-0.03		Asp144	0.48	0.23		Trp199		0.34
	Thr119	0.28	-0.05		Val145	0.48	0.23		His200		0.34

Differences for the average deuteration at the residue-level, $\overset{-}{\Delta D}$, between the apo-protein and the complexed protein with DNA, for some residues with known values (see S3 and S4 Figs).

**Fig 12:**
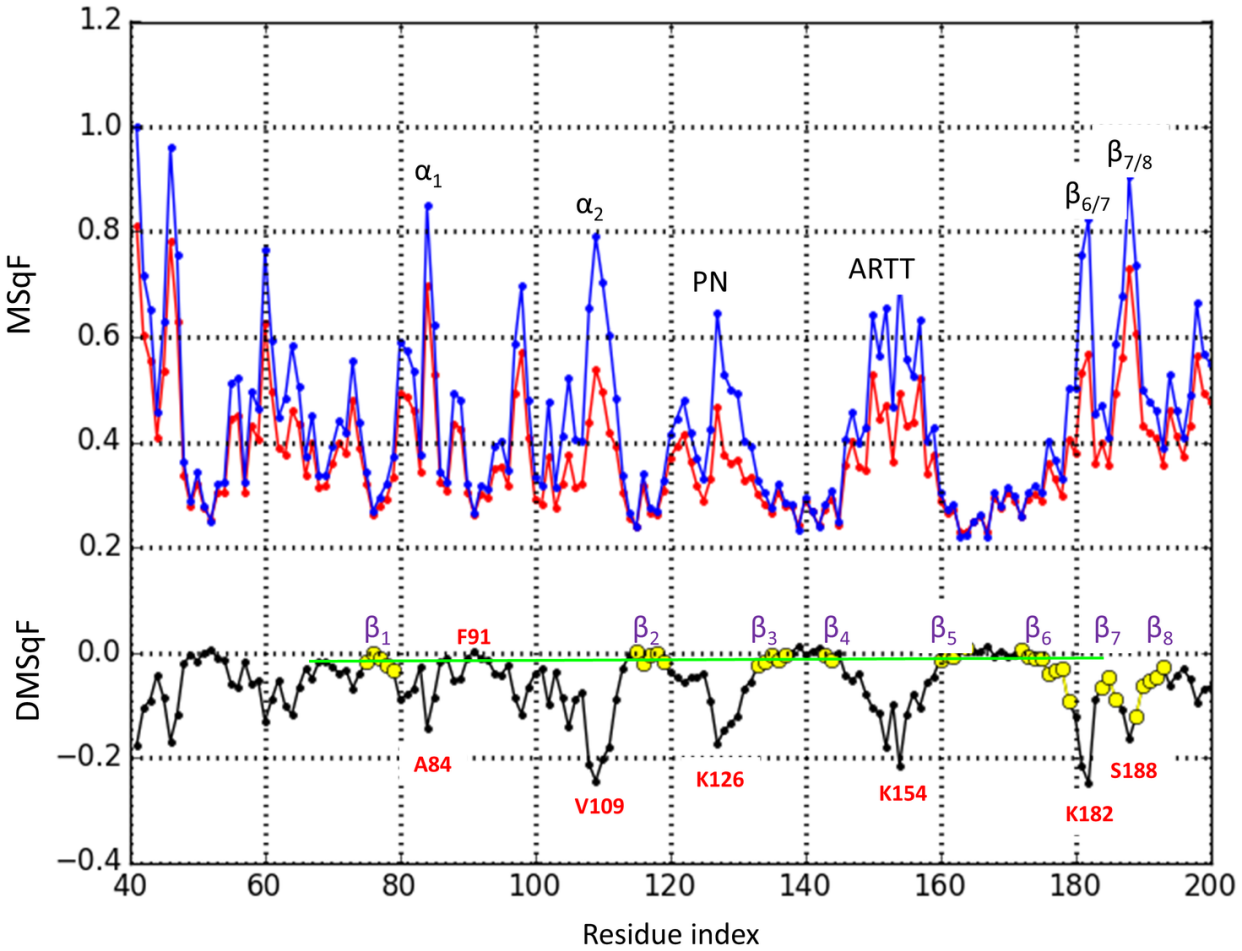
Scabin mobility in the Scabin· DNA complex. Normalized MSqF derived from a GNM (160 C_α_ nodes, γ = 1, cutoff = 10 Å) for the active conformation of Scabin in the ternary complex calculated without (blue) or with (red) the inclusion of the P-atoms of the DNA molecule as nodes. The difference (bound—apo) in relative mobility is plotted in black. Nodes corresponding to β-strands are highlighted in yellow.

The difference in the dynamics of Scabin between the apo and the bound form can be assessed by evaluating the MSqF profiles between the active, free form and the DNA-bound form, with both forms taken from the ternary complex model. For this calculation, a simple GNM elastic model was developed with a unique force constant and a unique cutoff distance. Fig 12 (upper panel) shows the profile of normalized and scaled profiles of fluctuations for both forms of the toxin. A difference-plot (lower panel) shows the relative variation in the fluctuations for the different structural elements and segments. As expected, the binding of DNA affects mainly those Scabin areas in direct contact with the ligand (*e*.*g*., α_2_, PN and ARTT loops, and the β_6/7_-turn). Moreover, the plot reflects variations in the average mobility of regions not in direct contact with the DNA molecule, but rather, those residues that are neighbors with the DNA-contacting residues. In effect, the reduction in mobility of the β_6/7_-turn (1^st^ β-turn of the SS_2_-loop) and the decrease in mobility of the β_7/8_-turn (2^nd^ β-turn of the SS_2_-loop), are both a result of the interaction with the dsDNA molecule and are compatible with experimental $\Delta \overset{-}{D}$ values. Also, the observed decrease in the $\Delta \overset{-}{D}$ level of the β_6_–β_8_ super-structure is compatible with the ternary complex model. Likewise, the GNM data predict an “induced” decrease in the SS_1_-loop mobility. If the β_2_ $\Delta \overset{-}{D}$ level is set as the baseline, the observed positive $\Delta \overset{-}{D}$ values for the coiled β_3/4_ segments agrees qualitatively with its predicted values.

## Discussion

The tendency of a protein in its native state to sample its “equilibrium dynamics” has been related to its ability to achieve its “biological function” [24]. In Scabin toxin, the conformational flexibility must be pertinent to the binding of the NAD^+^ and DNA substrates, to the hydrolysis of the glycosidic bond, to the coordination of the reactive species and transfer of the ADP-ribose intermediate to a guanine base and finally, to release the products of the reaction to begin a new cycle. A comprehensive description of the catalytic cycle requires an understanding of how the dynamics determine the function. However, to identify the dominant features of structural variability, the requirement is either experimental (X-ray or NMR models), or simulated (*e*.*g*. MD or Monte Carlo structures), or theoretical (*e*.*g*. all-atoms ANM decoys) collection of conformations. We have collected and deposited X-ray structures for the Scabin apo form (5DAZ), and structures of Scabin complexed with various competitive inhibitors of the NAD^+^ substrate (5EWK and 5EWY) and with the non-hydrolysable substrate analog, NADH (5TLB). A structural analysis from such a small number of “similar” structures has a limited scope and might lead to an incomplete or incorrect understanding of the toxin dynamics. In this sense, we employed MD simulations and GNM modeling to address the conformational variability of apo-Scabin in solution.

Thus, three 100 ns MD simulations of the Scabin macromolecule in explicit water were performed to sample the accessible conformational space of the apo protein in solution. An EDA performed on the combined (Grand) ensemble of MD conformations allowed us to identify the ‘*essential dynamics’* of the protein. The anharmonic components are illustrated in the large-scale motions observed in the projection of the conformations onto the lowest indexed PC_G_ modes and in their non-Gaussian density distributions.

The crystallographic temperature factors of C_α_-atoms for the X-ray structure were compared with the atomic fluctuations derived from the MD Grand ensemble. The relatively low correlation indicates intermolecular contacts that constrain the mobility of the crystallized protein. The high “thermal” fluctuations observed for of the SS_1_-loop and Asn110 in the crystal lattice is compatible with the idea that they are free of packing interactions. However, the X-ray fluctuations of the SS_1_-loop might also have their origin in the structural heterogeneity of the protein in this region. Indeed, alternative conformations of the Cys42 side-chain in the X-ray structure have been reported [14], which would negate the formation of the disulfide C42–C72 bridge and may produce an increased mobility for the whole SS_1_-loop. Accordingly, it was shown that ~9% of the apo-Scabin protein in solution contains Cys42 and Cys72 in a reduced state in the absence of any reducing agent [14].

To assess the MD of Scabin, a theoretical prediction of the protein dynamics based on an elastic model was implemented. Coarse-grained GNM was applied based on the *3D*-topology of the structure. This approach has been shown to accurately estimate atomic fluctuations in several systems when compared with the *B*-factor profiles [20]. However, better agreement has been found between calculated motions from elastic models and the structural variability from NMR decoys obtained in solution [25]. In effect, for the individual MD simulations, simple GNM models (uniform γ) could acceptably reproduce (*r* > 0.72) the MD MSqF in Scabin when the most anharmonic (lowest-indexed) PC modes were excluded. Notably, when the force constants were differentiated by motif to account for different connectivity and/or packing density, the correlation increased to *r* = 0.85 for the Grand ensemble. From all the above, all-atom MD simulations in explicit solvent can be considered the best approach to assess the dynamics of a protein in solution, and the PC modes of fluctuations derived from the Grand ensemble of MD conformations can be considered the best descriptors of both harmonic and anharmonic atomic fluctuations of apo-Scabin at thermal equilibrium; hence, they were used for further calculations.

The MD dynamics of Scabin were also assessed by H/D exchange. HDX is a powerful experimental technique that can provide direct information about protein dynamics and structural stability [26, 27] and therefore HDX data are usually employed to validate computational methods [23, 28]. The H/D measurements for apo-Scabin found for short (*L* ≤ 12) peptides are in overall agreement with the mobility profile observed in the Grand MD ensemble and the residue-based H/D probabilities obtained by the theoretical formulism. However, the theoretical, residue-level deuterium uptake probability, *p*_*i*_, offered the possibility of reporting deuterium levels for “missing” segments and those segments containing only medium and longer peptides, *e*.*g*., the L_b_-α_2_- L_c_ segment.

From the theoretical calculations, high probability of H/D exchange corresponds to highly mobile regions in loops and exposed, coiled structures, while secondary-structure elements exhibit a reduced tendency for H/D exchange [28]. Accordingly, the intrinsic propensity of different exposed segments/motifs for the H/D exchange follows this order: PN-loop > ARTT-loop > N_9_ > β_6/7_-turn > β_7/8_-turn > α_1_-α_2_. Within the α_1_-α_2_ motif, the peaks follow this order: Asp98(L_b_) > Lys94 (L_b_) > Ala84(α_1_) > Arg81(L_a_) > Leu108(α_2_). In proteins, regions of high mobilities are thought to be functionally important in substrate/ligand recognition [24]. Thus, considering that (*i*) dsDNA oligos as short as 10-base pairs, whose rod-like structure is highly probable, have been shown to bind Scabin and to accept the ADP-ribose moiety [9], and (*ii*) the transfer reaction requires a ternary complex where the reactive centers were in close proximity, then, it is reasonable that only those residues/motifs facing the *cis*-domain are considered as putative DNA binding sites. In summary, only the PN-loop, ARTT-loop, β_6/7_-turn, Lys94 (L_b_), Arg81(L_a_) and Leu108(α_2_) fulfill both criteria; consequently, it is highly probable they participate in binding the DNA substrate.

It is important to realize that the Scabin motifs/residues identified as DNA interacting areas from the MD/HDX exchange experiments correspond to those identified based on the ternary Scabin·NAD^+^·DNA model. Remarkably, the model of the ternary complex was obtained by an approach independent of the MD/HDX methodology—a “protein-protein” docking protocol based essentially on molecular mechanics. Moreover, the two methodologies were performed on two different systems: Scabin with solvent molecules and ions for the MD/HDX approach, while Scabin·NAD^+^ and DNA substrates in an implicit representation of the solvent were used for the second approach. Additionally, further direct and indirect evidence provide validation of the Scabin·DNA model, as follows.

### Assessment of the Scabin-DNA complex based on binding and activity data

Previously, three Scabin variants were reported with a significant impairment in the binding of the dsDNA substrate and showed this order in DNA affinity: Y129A (∞) < W128Y (9X) < W155A (3X), where the numbers indicate the impairment-factor with respect to the WT toxin [14]. In present work, the N110A variant was tested for its ability to bind the dsDNA substrate (1.5X reduction), to complete the study of the “Scabin-substrate bridge” (Asn110, Trp128, Tyr129, Trp155) and thus to assess the validity of the Scabin·NAD^+^·DNA model.

This result showed an absolute requirement of Tyr129 for the binding of a dsDNA oligonucleotide [14], which may be related to the specific location and nature of the Tyr129·DNA interactions according to the model—Tyr129 is the only residue that reaches into the minor DNA groove and contacts both DNA strands. In addition, the 7^th^ DNA_I_ base (5’→3’ direction) is exclusively bound by Tyr129 –all other interacting DNA bases share interactions with two or more residues. Consequently, only substitutions at Tyr129 (*e*.*g*. Y129A) can convert (in this case, G7 of DNA_I_) a functional DNA base into a non-functional binding center. Thus, the central location of this position on the innermost DNA strand seems to have a key role in DNA recognition by Scabin, and therefore, the lack of the Tyr129·G7 interaction might prevent a stable complex.

Trp128 is also relevant to binding of the DNA substrate by Scabin due to the ~10-fold decrease in DNA affinity of the W128Y variant [14]. The model is compatible with this result, since the pattern of Trp128·DNA_I_ interactions suggests that the W128Y substitution may preserve most of native interactions. Thus, the participation of Trp128 in DNA recognition may be underestimated when this variant is used as a reporter. Unfortunately, this could not be tested since the W128A variant was not stable.

The minor impairment (3X) in DNA binding by the W155A variant [14] also agrees with the nature of the Trp155 interactions depicted by the Scabin·DNA model—it shows only exposed van der Waals contacts with the DNA_I_ backbone. However, regarding the mART activity, Trp155 is absolutely required for the transferase activity towards both deoxyguanosine and guanine-containing dsDNA as nucleophilic substrates [14]. Based on the requirement of Trp155 for activity and on the well-established coordination role played by key ARTT-loop residue(s) in mART toxins, in general [18, 19, 29, 30], we proposed that Trp155 stabilizes the reactive guanine base for the transfer reaction–*i*.*e*., Trp155 interacts with the side-chain of the reactive guanine base [14]. This apparent discrepancy between the model and the activity data can be reconciled by considering that the model is an initial binding complex rather than a Michaelis-Menten complex, *i*.*e*., the active-site Scabin residues, interacting DNA bases, and configuration of the NAD^+^ substrate is likely not correctly positioned in the modeled complex for the reaction (*i*.*e*., the transition-state complex is not shown). Nevertheless, the ternary-complex model offers a plausible initial conformation for the reactive species which is compatible with X-ray binding complexes of other mART toxins [17, 30–32]. The center of the Trp155 indole ring is 7.8 Å from the center of the G9 base (DNA_I_), which may be the reactive base, and only 5.4 Å apart from the C-atom of the glycosidic ribose-nicotinamide bond. Incidentally, it has been reported that dsDNA oligonucleotides with centrally located guanine bases are optimal substrates for Scabin activity [9]. Interestingly, Lys154 is close to the major DNA groove and 6.8 Å from the G9 base. In a speculative sense, considering the flexibility, H-bond capability, and charged polar head of Lys154, its role in catalysis might be to disrupt the H-bond pattern of paired DNA bases, where a low dielectric cage—reinforced by Leu108 (α_2_)–enhances the electrostatic character of the “perturbing” interaction(s).

In summary, in the ternary complex model, Asn110 binds both substrates, which is compatible with the impaired binding of dsDNA (in the absence of NAD^+^) for the N110A Scabin variant. This finding supports the role of Asn110 in binding NADH (in the absence of DNA) as observed in the crystal structure. The minor effect of substitution with Ala on DNA affinity (1.5X) might reflect the small contribution of the H-bond of Asn110 to the binding energy. In fact, despite the direct interaction of Asn110 with NADH, there is a null effect on the GH activity for the N110A variant.

### Intrinsic dynamics of Scabin and the ligand binding mode

The most dominant GNM fluctuation mode in Scabin, which is the anticorrelated movement (coupled, but in opposite direction) of the N-lobe with respect to the C-lobe, corresponds to that reported for C3larvin toxin and is characteristic of the C2/C3-like topology [33]. Nevertheless, the secondary- and tertiary- structures of the regions that constitute both lobes are markedly different between C2/C3like and Scabin (*e*.*g*., α-lobe *v*. SS_1_-loop, respectively); moreover, the connection between both β-sheets is also different. In addition to the PN loop, Scabin connects both β-sheets by an α-loop-α super-secondary element (the α_1_-α_2_ motif).

Additionally, the deformation vectors that represent the ‘*essential dynamics’* of Scabin describe the displacement among these two domains as a clamping/release movement that resembles the “crab-claw” mechanism around the NAD^+^-binding pocket described previously for C3-like toxins [29, 33]. However, in Scabin, the inverse correlation of the α2 –PN distance with respect to the β6/7 –PN distance following the Φ_1_ direction is compatible with the idea of sequential binding of the substrates to constitute the ternary complex. This mechanism features NAD^+^ binding first followed by the DNA substrate (which does not imply that a bound NAD^+^ is a requirement for DNA binding). According to the previous statement and the model of the complex, the α_2_ and PN-loop approach each other to clamp the NAD^+^ substrate, while the PN loop and the β_6/7_-turn move further apart to make “room” in order to clamp the DNA_II_ strand by docking Tyr129 (PN loop) and basic residues at the β_6/7_-turn. Incidentally, the conformation of the Trp128-Tyr129 pair may be dependent on the ligation state of the toxin, since the Scabin·NADH complex is unable to bind a dsDNA oligonucleotide [14]. Simultaneously, the deformation of the α_1_-α_2_ motif exposes the α_2_, and an exposed α_2_ favors its docking into a major DNA groove in the Scabin *upper-region*. The null deuteration level observed at the N-terminal end of the L_b_-coil agrees with the “hinge” role of Phe91 in the relative movement of α_1_ with respect α_2_ –Phe91 is the center of a hydrophobic core that aids in keeping both β-sheets together.

### The Scabin·DNA complex in the context of mART toxin substrate binding

The role of ARTT and the PN loops in the interaction with the NAD^+^-substrate and the recognition of the target macromolecule has been well documented in most mART toxins [19, 29, 34]. The ARTT loop, except for the semi-conserved Trp155 (W_ARTT_) and Gln158 (Q_ARTT_), provide a unique sequence with non-conserved substitutions. The model and experimental data agree on the role of Scabin Trp155 in the binding of DNA. However, the DNA affinity in Pierisin-1 was not significantly affected in the W160A variant with respect to the WT protein [17]. Also, Trp155 is in close contact (< 4 Å) with NADH (5TLB) and NAD^+^ (this work) in Scabin, while Trp160 is farther (> 6Å) from the bound NAD^+^ in Pierisin-1 (5H6J). On the other hand, the unique substitutions involving Arg152 (consensus Ala) and Lys154 (consensus Pro) in Scabin are proposed to contact dsDNA and to be near the dsDNA, respectively (this work). All the above suggest an important role of the ARTT loop in Scabin and other members of the Pierisin-like group in binding both NAD^+^ and DNA substrates.

A Trp residue in the PN loop, W_PN_, is conserved in the Pierisin-like group. Notably, Trp128 in Scabin [14], and Trp127 in Pierisin-1 [17], are involved in the DNA and NAD^+^ substrate binding; therefore, it seems reasonable to extend this characteristic to the whole group. However, these two residues do not overlap spatially when the X-ray structures of Scabin (5TLB) and Pierisin-1 (5H6J) are superposed, and their pattern of interaction with the bound ligand (NADH and NAD^+^, respectively) are different between both complexes. In addition, Scabin harbors Lys130 as a basic residue in the PN loop, while 130-RNANR-134 (Pierisin-1 numbering) is a consensus motif in the PN loop of other members of the Pierisin-like group. Importantly, considering the structural flexibility observed in the PN loop among the different X-ray structures reported for Pierisin-1 [17], Lys130 in Scabin might be analogous to Arg134 in Pierisin-1. Nonetheless, while Lys130 is proposed to be a DNA interacting residue, neither Arg130 nor Arg134 in Pierisin-1 appear to be involved in the DNA binding [17]. Likewise, the Scabin α_3_ helix (123-DLYKT) differs from the consensus -RNKKK sequences in the group. Of these elements, only Thr127 is proposed as a DNA-interacting residue in Scabin, although it may not be catalytically relevant since it interacts with two bases that are contacted by other residues. On the contrary, two Lys residues of the positively-charged motif in Pierisin-1 have been shown to be required for DNA binding [17]. Taken together, these observations suggest that the PN-loop of Scabin has a unique mode for interacting with the DNA substrate, which is distinct from the other Piersin-like toxins.

Regarding the L_a_ segment, Ser80 in Scabin interacts with NAD^+^ (this work) and with NADH [14], but does not seem to interact with DNA (this work). Notably, the opposite occurs in the other members of the Pierisin-like group with the semi-conserved Arg substitution; in Pierisin-1, for example, Arg73 does not interact with NAD^+^ (5H6J) but does with DNA [17]. However, the contiguous position is occupied by a conserved Arg in the group, R_La_, which seems to exhibit the same characteristic in all group members as Arg81 in Scabin (5TLB) and Arg74 in Pierisin-1 (5H6J) has been reported to interact with NADH/NAD^+^, but none of them with DNA (this work and [17]). The L_a_ segment is the topological equivalent to the α_5a_ helix in C2/C3-like toxins, whose residues interact with the bound NAD^+^.

The α_2_ helix harbors the 104-ES(YV)LV sequence in Scabin, while the consensus is the -YG(FA)KN sequence for the rest of the toxins in the group (the residues in the parenthesis are conservative substitutions and face the interior of Scabin and Pierisin-1, respectively). The other residues, exposed to the aqueous solution, confer a negative → neutral electrical polarity to α_2_ in Scabin (following the N→C direction), while imparting a neutral → positive electrical polarity to α_2_ in Pierisin-1 (and other members of the group). The hydrophobic C-terminus of α_2_ is herein proposed to interact with the hydrophobic major groove of dsDNA, where the charged or polar C-terminus on the other member of the group (consensus -KN) would be unfavorable. Incidentally, the equivalent helix in Pierisin-1 (named α_E_) is not thought to be a DNA-interacting motif [17]. On the contrary, and despite some non-conserved substitutions, the polarity of the α_1_ in Scabin (α_D_ in Pierisin-1, [17]) is similar in all the members of the group: neutral → negative dipole. Importantly, α_1_ is not proposed to be a DNA-interacting motif in any of the Pierisin-like group members.

Contiguous to α_2_, an Asn residue in the L_c_ segment, N_Lc_, is conserved in the Pierisin-like group. Asn110 interacts with NADH (5TLB) and NAD^+^ in Scabin, and Asn109 interacts with NAD^+^ in Pierisin-1 (5H6J), highlighting its clear role in binding the NAD^+^ substrate. However, Asn110 also interacts with the DNA substrate in Scabin, while Asn109 is not proposed to interact with DNA in Pierisin-1 [17]. The L_c_ segment is topologically equivalent to α_2_ of the helical N-lobe in the C2/C3-like toxins, α-lobe. Therefore, the α_1_-α_2_ motif (L_a_-α_1_-L_b_-α_2_-_Lc_) is a segment with mixed character in terms of its participation in the binding of both substrates when Scabin is compared with other members of the Pierisin-like group.

In Scabin, the β_6/7_-turn protrudes and includes a unique cluster of basic and HB-donor residues, the 180-KKTR sequence, proposed to interact electrostatically with the DNA. In Pierisin-1, the equivalent β-turn harbors Arg187 which is required for DNA binding [17]. Consequently, we propose that these basic residues favor the exploration and eventual binding of the DNA substrate in these toxins. Notably, this turn is topologically equivalent to the β_7/8_-turn (region IV) in C3cer toxin, for example, which has been reported to be an interacting element with its protein target [35]. However, Arg81 in Pierisin-1 is a residue that superposes with Cys176 in Scabin (structural alignment). Arg81 is centrally located in β_6_ and faces the *trans* side of these proteins and is an essential requirement for DNA binding by Pierisin-1 [17]. Hence, although these proteins present a positively charged cluster of residues involved in DNA binding, the offset in the sequence and difference in the *3D* location of these residues between Scabin and Pierisin-1 reveal their distinct roles in the binding of the DNA substrate.

In summary, the conserved R_β1_ (Arg77) and R_La_ (Arg81) residues clearly participate in the binding of NAD^+^ (and/or catalysis), as observed in both Scabin and Pierisin-1, and other mART toxins/proteins. In Scabin, but not in Pierisin-1, N_Lc_ (Asn110), W_ARTT_ (Trp155), and Q_ARTT_ (Gln158) contact both the NAD^+^ as well as the DNA substrates. Notably, W_PN_ contacts both substrates in Scabin (Trp128) and in Pierisin-1 (Trp127), despite the different constitution and configuration of the PN-loop. Moreover, W_PN_ is the only Trp residue absolutely conserved in the group, even appearing in Pierisin-medi and CARP-1 proteins.

### Conclusions

In the present work, we combined experimental (HDX), simulated (MD), and theoretical (GNM and ΔG_i,HDX_) approaches to systematically study the dynamics of apo-Scabin, and to assess the Scabin·NAD^+^·DNA model. MD simulations revealed that the apo-protein folded solution structure does not significantly deviate from the crystal structure, beyond the conformation of the exposed, mobile regions. GNM and EDA analyses allowed characterization of the ‘*essential dynamics*’ of the toxin in its crab-claw-like mechanism of two topological domains. The “crab-claw” motion resembles the dominant motions of C3-like toxins and emerges as an intrinsic property of the conformation of the common, central β-scaffold of the catalytic single-domain mART toxins. The exposure and high mobility of the motifs harbored at the *cis* side of this β-core in Scabin (helix α_2_, β_6/7_-turn, etc.) suggest they are involved in the recognition and binding of the DNA substrate.

A ternary Scabin·NAD^+^·DNA model was obtained by an independent docking methodology, where the nature of the intermolecular interactions is polarized in an “electrostatic” *lower-area* and “hydrophobic” *upper-area*. The interactions in the *middle-area* are mixed in character, with both substrates bridged by the participating residues. In this area, and because of its distinctive location, extent, and nature of the interactions, Tyr129 plays a major role in DNA binding.

Thus, considering (*i*) the correlation between the putative DNA interacting sites based on pure “dynamic” criteria evaluated on the apo form of Scabin, and the actual DNA interacting sites obtained by statistical evaluations of interaction energy and shape complementarity with the Scabin·NAD^+^ model, (*ii*) the agreement between structural, energetic, and dynamic features of the model with biochemical (binding and activity) and dynamic (HDX) data, and (*iii*) the fact that 4 residues that bind both substrates are conserved or semi-conserved in the Pierisin-group, then the proposed model is feasible.

Based on current data on the Pierisin-like mART toxin group, the sequence motif R_β1_–R_La_–N_Lc_–STT_β2_ –W_PN_–W_ARTT_–(QxE)_ARTT_ emerges as a signature motif involved in the mART activity of these proteins/toxins. Nevertheless, since (*i*) the reported DNA-contacting residues/motifs in Pierisin-1 are not present in Scabin, and (*ii*) most of the proposed DNA-contacting residues/motifs in Scabin are not present in other members of the Pierisin-like group, then it appears that Scabin exhibits a unique DNA-binding motif within the Pierisin-like group (Table 3). Finally, the binding capability of Lys130 and Leu108, and the potential catalytic role of Lys154 are outcomes of the ternary complex model and are definite targets for future investigation.

**Table 3:** Key residues in the interaction of Scabin with NAD^+^ and DNA.

Element	Res	DNA	NAD^+^	Frequency
β_1_	Arg77		+	conserved
L_a_	Ser80		+	unique
	Arg81		+	conserved
L_b_	Gln100	+		unique
	Asp102	+		consensus
α_2_	Glu104	+		unique
	Ser105	+		unique
	Leu108	+		unique
	Val109	+		unique
L_c_	**Asn110**	+	+	conserved
	Gln111	+		unique
β_2_	Ser117		+	conserved
	Thr119		+	conserved
PN-loop	Thr127	+		unique
	**Trp128**	+	+	conserved
	Tyr129	+		unique
	Lys130	+		unique
ARTT-loop	Arg152	+		unique
	**Trp155**	+	+	conserved
β_5_	**Gln158**	+	+	conserved
	Glu160		+	conserved
β_6/7_	Lys180	+		unique
	Lys181	+		Unique
	Arg183	+		Unique

Scabin residues proposed to interact with dsDNA and/or with the NAD^+^ substrate. Residues that bind both substrates in the ternary complex model are shown in bold. The apparent frequency for motifs/elements in the Pierisin-like group are shown, according to the primary sequence alignment. Semi-conserved refers to conserved residues in all members, except for MTX toxin from *L*. *sphaericus*.

## Materials and methods

### Materials

Unless otherwise stated, materials were purchased from Sigma-Aldrich.

### Scabin expression and purification

The expression and purification methods for Scabin toxin were conducted as described previously [9]. *GH activity*–Scabin (WT 50 nM; N110A 1 μM) was incubated with increasing concentrations of ε-NAD^+^ (0–500 μM) in GH buffer containing 20 mM Tris-HCl, pH 7.9, and 50 mM NaCl. Reactions were performed in triplicate on a Cary Eclipse fluorescence spectrophotometer (Varian Instruments, Mississauga, Canada) and monitored for 20 min. The slope based on an ε-AMP standard curve (arbitrary units μM^-1^) was used to calculate the steady rate of formation of ε-ADP-ribose (μM·min^-1^) from the rate (arbitrary units·min^-1^). Measurements were conducted in triplicate and the calculated GH activity was plotted against initial ε-NAD^+^ concentration and fit to a hyperbolic function (Michaelis-Menten model). Excitation and emission wavelengths were set to 305 and 405 nm, respectively, with band-passes of 5 nm.

### Cyanine-3-DNA binding

Synthetic dsDNA oligomers (oligomer 1: 5’-GGAAGAGAGAGAGAAAGAGAG-3’; oligomer 2: 5’-CTCTCTTTCTCTCTCTCTTCC-3’) with a 5’ cyanine-3 (Cy-3) tag on oligomer 1 were purchased from Sigma-Aldrich. The oligomers were mixed in equal molar amounts and annealed by heating to 90°C followed by cooling to 20°C at a rate of 1°C/min in a Techne TC-512 PCR (Burlington, NJ) to make a blunt ended substrate (Cy3-dsDNA). In an ultra-micro quartz cuvette (3 mm x 3 mm), 5 μM of Cy3-dsDNA in 25 mM Tris-HCl, pH 8.2, 100 mM NaCl was titrated with increasing concentrations of either wild-type Scabin or the N110A variant, and the change in anisotropy was measured. Data were collected in a ‘T-format’ configuration using a PTI-Alphascan-2 spectrofluorimeter (Photon Technologies Inc., South Brunswick, NJ) equipped with a thermally controlled cell holder (22°C). The binding constants were calculated as described previously [9].

### Microfluidic chip fabrication

The microfluidic chip was made on a poly (methyl methacrylate) (PMMA) substrate with dimensions of 5.1 cm × 2.0 cm × 1.2 cm and the proteolytic chamber was etched onto the chip using a VersaLaser engraver (Universal Laser, Scottsdale, AZ) as previously described [36]. The time-resolved electrospray ionization (TRESI) mixer was made by inserting a glass capillary (152 μm outer diameter) into a metal capillary (inner diameter 178 μm) to create an intercapillary space of 26.8 μm. The end of the glass capillary was sealed using the VersaLaser and a notch was cut 2 mm from the sealed end. For reaction quenching, a T-mixer with a dead volume of 51 nL was used to mix glacial acetic acid with deuterated protein. Pepsin-agarose beads were crosslinked as previously described [37]. The proteolytic chamber was filled with pepsin-agarose beads, and a 33G metal capillary was used as an outlet to the electrospray ionization source. Hamilton syringes were used to deliver reagents through glass capillaries using Pump 11 Elite infusion pumps (Harvard Apparatus, Holliston, MA). The device was placed at the front end of a Synapt G1 mass spectrometer (Waters, Mississauga, ON) for HDX-MS experiments.

### Hydrogen-deuterium exchange of Scabin

The HDX reaction took place inside the time-resolved electrospray ionization (TRESI) mixer. Either 35 μM Scabin alone or 35 μM Scabin with 140 μM DNA (forward strand: 5’-GGAAGAGAGAGAGAAAGAGAG-3’) in 500 mM ammonium acetate, pH 8.2 was flowed through the inner glass capillary (flow rate 1 μL/min) and deuterium was passed through the outer metal capillary (flow rate 1 μL/min). Total flow rate into the TRESI mixer (2 μL/min), along with intercapillary space and with the glass capillary pulled back 5 mm, was used to calculate a mixing volume of 124 nL and a reaction time of 4.14 s. The instrument was operated with a source voltage of 3000 V in positive-ion mode, and samples were scanned over a range of *m/z* 400–1500. Apo-Scabin HDX was performed using four biological replicates each having four technical replicates; ligand HDX experiments were performed using 3 biological replicates, each containing four technical replicates. A pepsin spectrum was collected to eliminate peptides within the Scabin digestion profiles that correspond to self-digestion of pepsin. A Scabin digestion profile was collected in the absence of D_2_O to identify peptides. In the cases where peptides were overlapping, spectra were collected using ion-mobility spectrometry to separate peptides based on their size, shape and charge.

### Hydrogen-deuterium exchange data analysis

Digested peptides were identified using mMass [38] and corresponding masses were analyzed using the FindPept tool on the ExPASy Proteomics server (mass tolerance ± 0.5 Da) to determine peptide sequences. Peptides that could not be confirmed due to multiple ‘high-certainty’ hits (ΔM ± 0.5 Da) in FindPept were subjected to MS/MS sequencing to confirm identification. The experimental deuterium uptake was calculated using an in-house-developed FORTRAN software (Wilson, D.J., unpublished) that models the change in the isotopic distribution to determine the percent uptake of deuterium for each peptide of interest [39].

### Force field setting and structure preparation

Protein preparation and molecular mechanics (MM) calculations were performed using the computational suite Molecular Operative Environment (MOE) release 2016.08 (Chemical Computing Group Inc, Montreal, CA). The force field employed was the AMBER10 parameters set (ff10). For the implicit solvent model, the Generalized Born-Volume Integral (GB-VI) formulism was employed, with dielectric *ε*_*pro*_ = 2 for the interior of the protein and *ε*_*sol*_ = 80 (water) for the exterior. A switching, cutoff function was defined between 10 and 12 Å for non-bonded interactions. The MOE Protonate3D module was used to assign the ionization states and tautomers of side-chains of the apo-Scabin X-ray structure (5DAZ) at *T* = 300 K, pH = 7.4 and 0.1 M of ionic strength, along with the GB-VI solvation model and MMFF94 partial charges. Backbone atoms were fixed, and the system was energy minimized (RMSG ≤ 0.001 kcal/mol/Å^2^).

### Molecular dynamics simulations

The MOE Solvate module was used to locate the center of mass of the toxin at the center of a periodic box of 61.56 x 58.99 x 58.86 Å^3^ (edge lengths). The energy function was updated to vacuum (*ε*_*sol*_ = 1), the charges were neutralized by incorporating 5 K^+^ ions at optimal locations, and the system was solvated by keeping the crystallographic waters for a total of 6197 TIP3P water molecules (at 1.023 g/cm^3^ density). The system was energy minimized in a stepwise fashion (each to a RMSG ≤ 0.01 kcal/mol/Å^2^) as follows: first, the protein was fixed and the solvent (water and ions) was relaxed, then backbone atoms were fixed, and side-chains were energy minimized; finally, the full system was energy minimized. With this molecular system, three 105 ns molecular dynamics (MD) simulations were performed by the Scalable Molecular Dynamics (NAMD) simulator release 2.9 [40] under periodic boundary conditions by wrapping protein and solvent, with an integration time of 1 fs, and rigid water, following the sequential steps: (*i*) 1000 ps of heating from 0 to 295 K, with heavy atoms tethered to a standard deviation of *r* = 0.5 Å; (*ii*) 1000 ps equilibration at 295 K and *r* = 1.0 Å; (*iii*) 1000 ps of equilibration at 295 K and *r* = 2.0 Å; (*iv*) 2000 ps of equilibration at 295 K and *r* = 4.0 Å, and finally (*v*) an NVT ensemble of production phase at 295 K for 100 ns, recorded each 5 ps.

### Modeling the Scabin·NAD^+^·DNA complex

Using the Scabin·NADH X-ray structure (5TLB) stripped from CWMs, the NADH molecule was crafted *in situ* to NAD^+^. The force-field MOE Amber12: EHT was used to parameterize the NAD^+^ molecule. The Protonate3D protocol was performed over the whole system but protecting the oxidized state of the ligand. In the new system, the NAD^+^ molecule (10 kcal/mol, 0.25 Å buffer) and the heavy-atoms (100 kcal/mol), except side-chains with atoms at ≤ 4.5 Å from the nicotinamide moiety, were re-packed by conducting an energy minimization until a RMSG ≤ 0.001 kcal/mol/Å^2^ was achieved. The 21-base pair double stranded DNA oligo, 5’-GGAAGAGAGAGAGAAAGAGAG-3’ (forward strand), was built in a B-helix conformation. A coarse-grained MOE protein-protein docking was performed using the dsDNA molecule as a substrate onto the Scabin·NADH (receptor). This approach considers rigid side-chains and an implicit solvent model. The 30 highest-ranked ternary decoys were further optimized with an iterative protocol implemented by alternating a “rigid-body” energy minimization (RMSG ≤ 0.1 kcal/mol/Å^2^) of the DNA molecule onto a fixed Scabin·NAD^+^ complex *with* a “conformational” energy minimization (RMSG ≤ 0.01 kcal/mol/Å^2^). This was conducted by relaxation of free interfacial (loop atoms and side-chains in contact with DNA) and restrained with 10 kcal/mol neighbours (≤ 4.5 Å from the interfacial atoms) Scabin·NAD^+^ atoms onto a fixed DNA molecule, until the difference in the total energy of the system between two consecutive iterations was lower that 0.1 kcal/mol. The decoy with the lowest total energy was saved as the Scabin·NAD^+^·DNA *ternary complex model*.

### Structure analysis

All the structure dynamics calculations were performed in the Protein Dynamics (ProDy) package release 1.5.1 [41] using Python 2.7.9 (Python Software Foundation, Delaware, USA). The Normal Mode Wizard (NMWiz) release 1.2 was plugged into the Visual Molecular Dynamics (VMD) Viewer, release 1.9.2 [42]. For the essential dynamic analysis (EDA), and for the Gaussian network model (GNM), only *N* = 160 C_α_-atoms (Ala41–His200) were considered for the full-length protein, omitting the 5 N-terminal residues because of the high mobility in this region.

For the optimized C_α_ superposition of the ensemble configurations of a set of *N* atoms, ProDy implements an iterative superposition (“*interpose”*) that gives a unique solution that minimizes the average RMSD of the ensemble. Briefly, the *i*^th^ member of the ensemble, *i*.*e*. the *i*^th^ conformation, is represented by a *3N*-dimensional column vector defined as

$$R_{i}={\left[r_{1,i}{\;r}_{2,i}\;r_{3,i}\ldots r_{N,i}\right]}^{T}$$ (2)

with ***r***_*p*,*i*_ = [*x*_*p*_ *y*_*p*_ *z*_*p*_]_*i*_ being the *3D*-position vector of the *p*^th^ atom in the *i*^th^ configuration. For an ensemble of M configurations, the average *3N*-dimensional column vector is defined as

$$\bar{\text{R}}=\frac{1}{M}\sum_{i=1}^{M}{\text{R}}_{i}={\left[\bar{{\text{r}}_{1}}\;\bar{{\text{r}}_{2}}\;\bar{{\text{r}}_{3}}\ldots \bar{{\text{r}}_{p}}\right]}^{\text{T}}$$ (3)

The mean square fluctuation (MSqF) and the sum of square fluctuations (SSqF) for the *p*^th^ position are calculated over all the *M* conformations by

$$MSqF_{p}=\langle \Delta r_{p}{}^{2}\rangle =\frac{1}{M}\sum_{i=1}^{M}{\left(r_{p,i}-\bar{r_{p}}\right)}^{2}$$ (4)

$$SSqF_{p}=\sum_{i=1}^{M}{\left({\text{r}}_{p,i}-\bar{{\text{r}}_{p}}\right)}^{2}$$ (5)

The root means square deviation (RMSD) of the *i*^th^ configuration is calculated over all the *N* atoms by

$$RMSD_{i}={\langle \Delta r_{i}{}^{2}\rangle }^{1/2}=\sqrt{\frac{1}{N}\sum_{p=1}^{N}{\left({\text{r}}_{p,i}-\bar{{\text{r}}_{p}}\right)}^{2}}$$ (6)

The average RMSD for the ensemble is calculated by

$$\bar{RMSD}=\langle RMSD_{i}\rangle =\frac{1}{M}\sum_{i=1}^{M}RMSD_{i}$$ (7)

The RMSD between two *i* and *j* conformations are calculated by

$$RMSD_{ij}={\langle \Delta r_{ij}{}^{2}\rangle }^{1/2}=\sqrt{\frac{1}{N}\sum_{p=1}^{N}{\left({\text{r}}_{p,i}-{\text{r}}_{p,j}\right)}^{2}}$$ (8)

The interpose routine proceeded as follows: (*i*) a random configuration of the ensemble was selected, and its coordinates were taken as an *average* conformation of the ensemble; (*ii*) each member of the ensemble was superimposed onto the average conformation by a rigid-body translation and rotation to minimize the RMSD of the configuration (Eq 5); (*iii*) a new average conformation was calculated for the ensemble by using Eq 2. Steps (*ii*) and (*iii*) were iteratively performed until the RMSD between two successive average configurations (Eq 7) were lower than an arbitrary threshold (usually 0.001 Å).

### Theoretical calculation of the residue-level deuteration in Scabin

In the original work of Bahar and colleagues [24], they demonstrated that for a series of proteins, a good correlation exists between the free energy change per unit of deformation, $\Delta G_{i,GNM}/\Delta R_{i}^{2}$, calculated by means of the [Γ^-1^]_*ii*_ (the *i*^*th*^ diagonal element of the inverse of the Kirchhoff matrix) in GNM and the experimental free energy change associated with the deformational process of opening-closing that exposes a backbone amide proton for exchange, Δ*G*_*i*,*HDX*_. Eq 7 in Bahar (1998)[24] can be rewritten as

$${\Delta G}_{i,HDX}\propto \frac{{\Delta G}_{i,GNM}}{{\Delta R}_{i}^{2}}\propto \frac{1}{{\left[\Gamma^{-1}\right]}_{ii}}$$ (9)

where Δ*G*_*i*,*HDX*_ corresponds to the standard free-energy change of the conformational backbone N-amide *close* ↔ *open* equilibrium, according to

$${\Delta G}_{i,HDX}=-{RTln\frac{r_{c⟶o}}{r_{o⟶c}}|}_{i}=-RTln(K_{i})$$ (10)

with *r*_*i*_ representing the rate constant of the unidirectional transitions, and 0 < *K*_*i*_ ≤ ∞ is the equilibrium constant for the transition of the *i*^*th*^ amide N-atom. From Eq 9, the relative free-energy change, Δ*G*_*i*,*HDX*_ − Δ*G*_*min*,*HDX*_, can be shown as

$$\Delta \Delta G_{i,HDX}=-RTln\left(\frac{K_{i}}{K_{max}}\right)=RTln\left(p_{i}\right)$$ (11)

with $0<K_{i}^{'}\leq 1$. Thus, $K_{i}^{'}$ might be considered as the relative probability, *p*_*i*_, of the *i*^*th*^ residue that exchanges its amide proton with respect to the most exposed/mobile—always exchangeable—proton (with *p*_*i*_ = 1).

### Plotting and rendering

Unless otherwise stated, plots were performed by the Matplotlib library running in Python 2.7.9, and protein rendering was performed using the computational suite Molecular Operative Environment (MOE). The *molecular surfaces* are solvent-excluded surfaces obtained by rolling a probe sphere of 1.4 Å diameter (water radius) and colored by several schemes.

## Supporting information

S1 Fig Density distributions of MD conformations of apo-Scabin. Density distributions of 30,000 conformations of the Grand ensemble of conformations binned according to their projected values onto (**a**) PC_G_(1), (**b**) PC_G_(2), and (**c**) PC_G_(15) principal components. In all, the distributions are colored according to the original trajectory (Fig 1) as Run_a_ in blue, Run_b_ in green, and Run_c_ in red.
(PDF)

S2 Fig Crystal contacts in apo-Scabin. Ribbon representation of apo-Scabin (PDB: 5DAZ) X-ray structure showing in green sectors that contact neighbor (≤ 4.5 Å) molecules in the crystal lattice. Asn110 and the SS_1_-loop are in light brown to highlight the absence of crystal interactions.
(PDF)

S3 FigDeuteration of apo-Scabin.***Lower panels***. Deuteration level, *D*_*v*_, of 53 Scabin peptides with length between 3 and 12 residues. The two C-terminal (striped) and proline (“-”) residues of each peptides were excluded in the calculation as indicated in the *Materials and Methods*. ***Upper panels***. Residue ID, Residue index, and average deuteration level at the residue-level, ${\overset{-}{D}}_{i}$. In both cases, values spectrally colored from blue (low)–green (medium)–red (high).(PDF)

S4 FigDeuteration of Scabin complexed with DNA.***Lower panels***. Deuteration level, *D*_*v*_, of 23 Scabin peptides with lengths between 3 and 12 residues in the presence of DNA. The two C-terminal (striped) and proline (“-”) residues were included in the calculation as indicated in the *Materials and Methods*. ***Upper panels***. Residue ID, Residue index, and average deuteration level at the residue-level, ${\overset{-}{D}}_{i}$. In both cases, values spectrally colored from blue (low)–green (medium)–red (high).(PDF)

## Abbreviations

ANM: anisotropic network model
ARTT: ADP-ribosylation turn-turn
CWM: crystallographic water molecule
EDA: essential dynamic analysis
EHT: extended Hückel theory
GB-VI: generalized Born-volume integral
GH: glycohydrolase
GNM: Gaussian network model
H/D: hydrogen-deuterium
HDX: hydrogen-deuterium exchange
mART: mono-ADP-ribosyltransferase
MD: molecular dynamics
MM: molecular mechanics
MMFF94: Merck molecular force-field mmff94
MSqF: mean square fluctuation
PARP-1: poly-ADP-ribosylpolymerase 1
PC: principal component
PDB: Protein Data Bank
PN: phosphate-nicotinamide
RMSD: root mean square deviation
RMSG: root mean square gradient
SSqF: sum of square fluctuation
