# Supplementary figures and images for: Dynamics of Scabin toxin. A proposal for the binding mode of the DNA substrate
Source: PLoS One. 2018 Mar 15;13(3):e0194425. doi: 10.1371/journal.pone.0194425 (PMC5854381; doi:10.1371/journal.pone.0194425)

**Fig. S1**

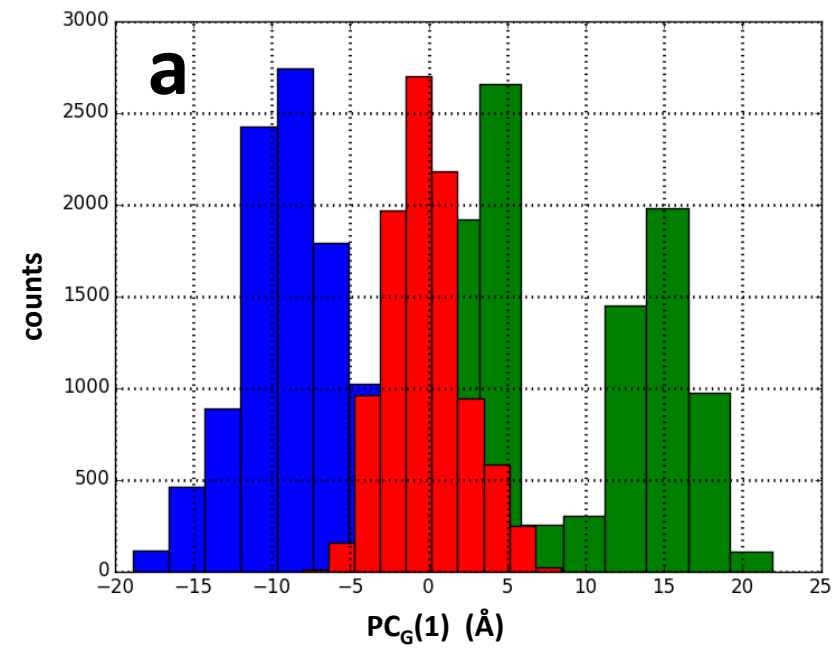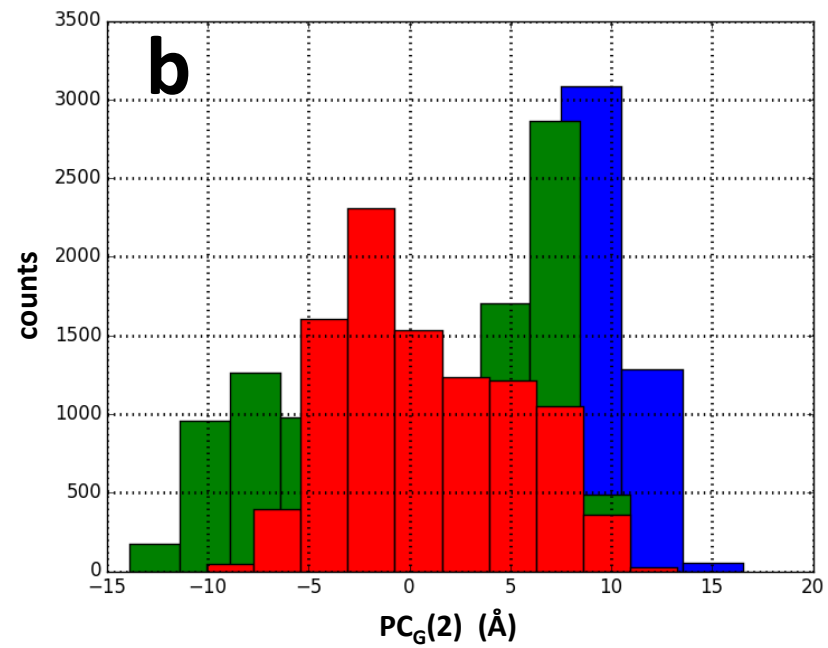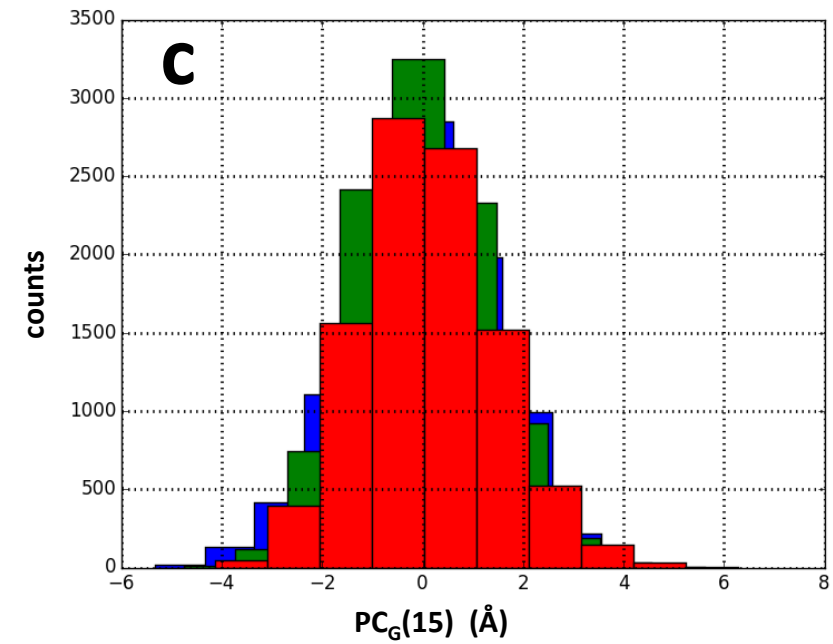

Supplement: S1 Fig — Density distributions of 30,000 conformations of the Grand ensemble of conformations binned according to their projected values onto (a) PCG(1), (b) PCG(2), and (c) PCG(15) principal components. In all, the distributions are colored according to the original trajectory (Fig 1) as Runa in blue, Runb in green, and Runc in red. (PDF) [file pone.0194425.s001.pdf]

**Fig. S2**

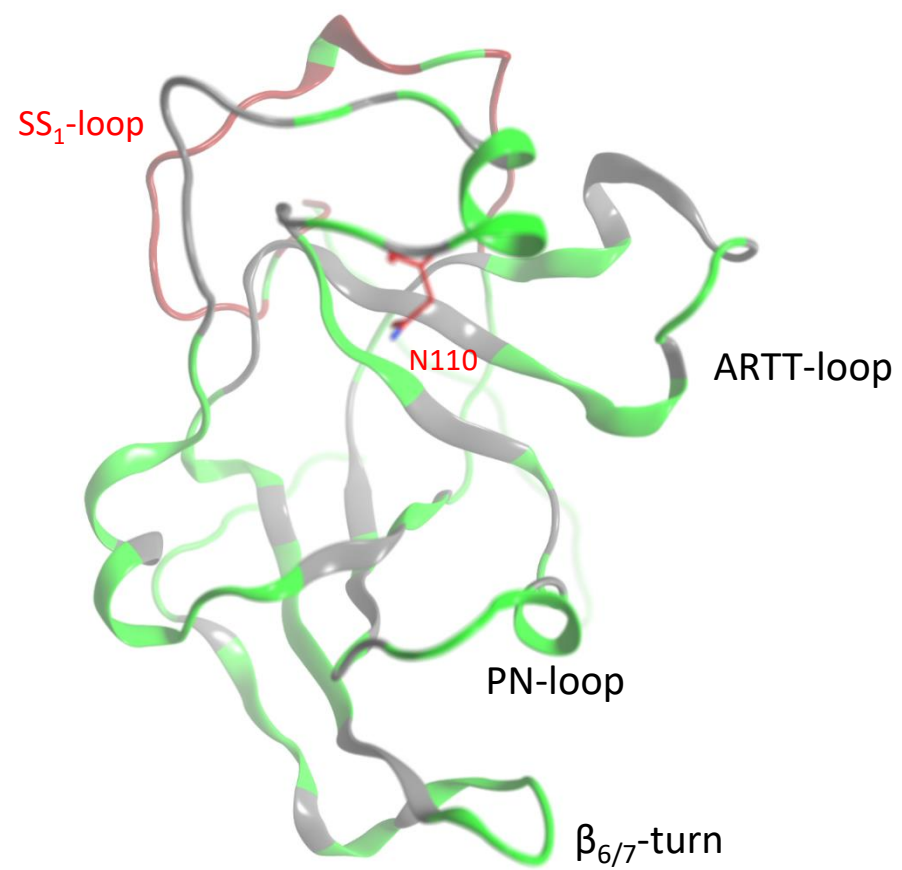

Supplement: S2 Fig — Ribbon representation of apo-Scabin (PDB: 5DAZ) X-ray structure showing in green sectors that contact neighbor (≤ 4.5 Å) molecules in the crystal lattice. Asn110 and the SS1-loop are in light brown to highlight the absence of crystal interactions. (PDF) [file pone.0194425.s002.pdf]

**Fig. S3**

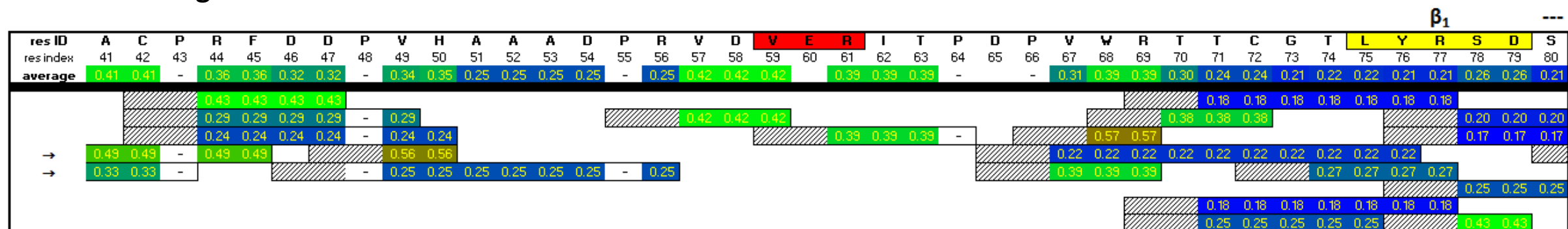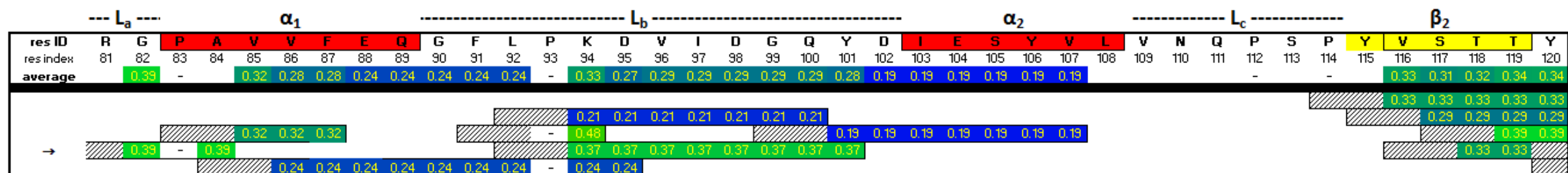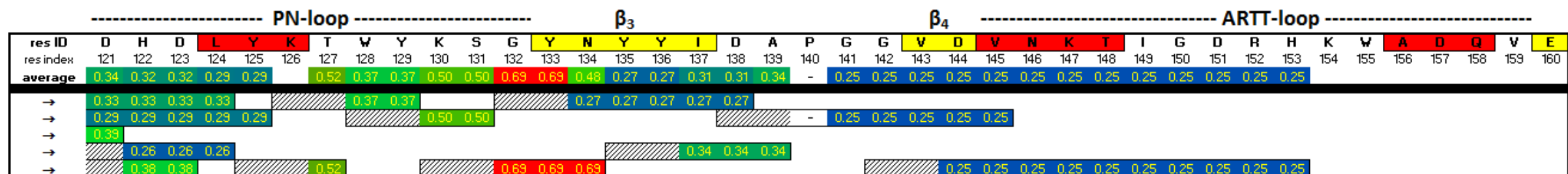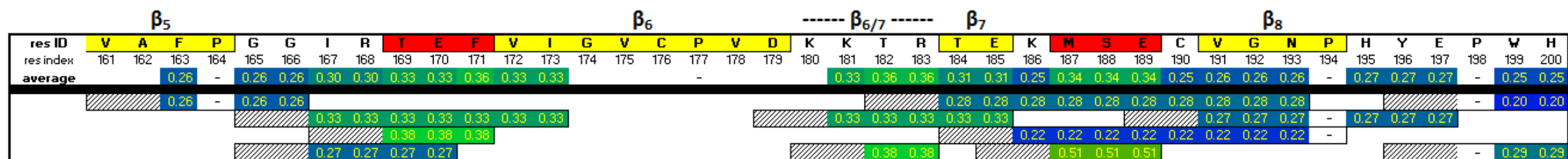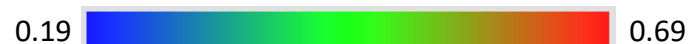 $D_v$  or  $\bar{D}_i$

Supplement: S3 Fig — Lower panels. Deuteration level, Dv, of 53 Scabin peptides with length between 3 and 12 residues. The two C-terminal (striped) and proline (“-”) residues of each peptides were excluded in the calculation as indicated in the Materials and Methods. Upper panels. Residue ID, Residue index, and average deuteration level at the residue-level, D-i. In both cases, values spectrally colored from blue (low)–green (medium)–red (high). (PDF) [file pone.0194425.s003.pdf]

Fig. S4

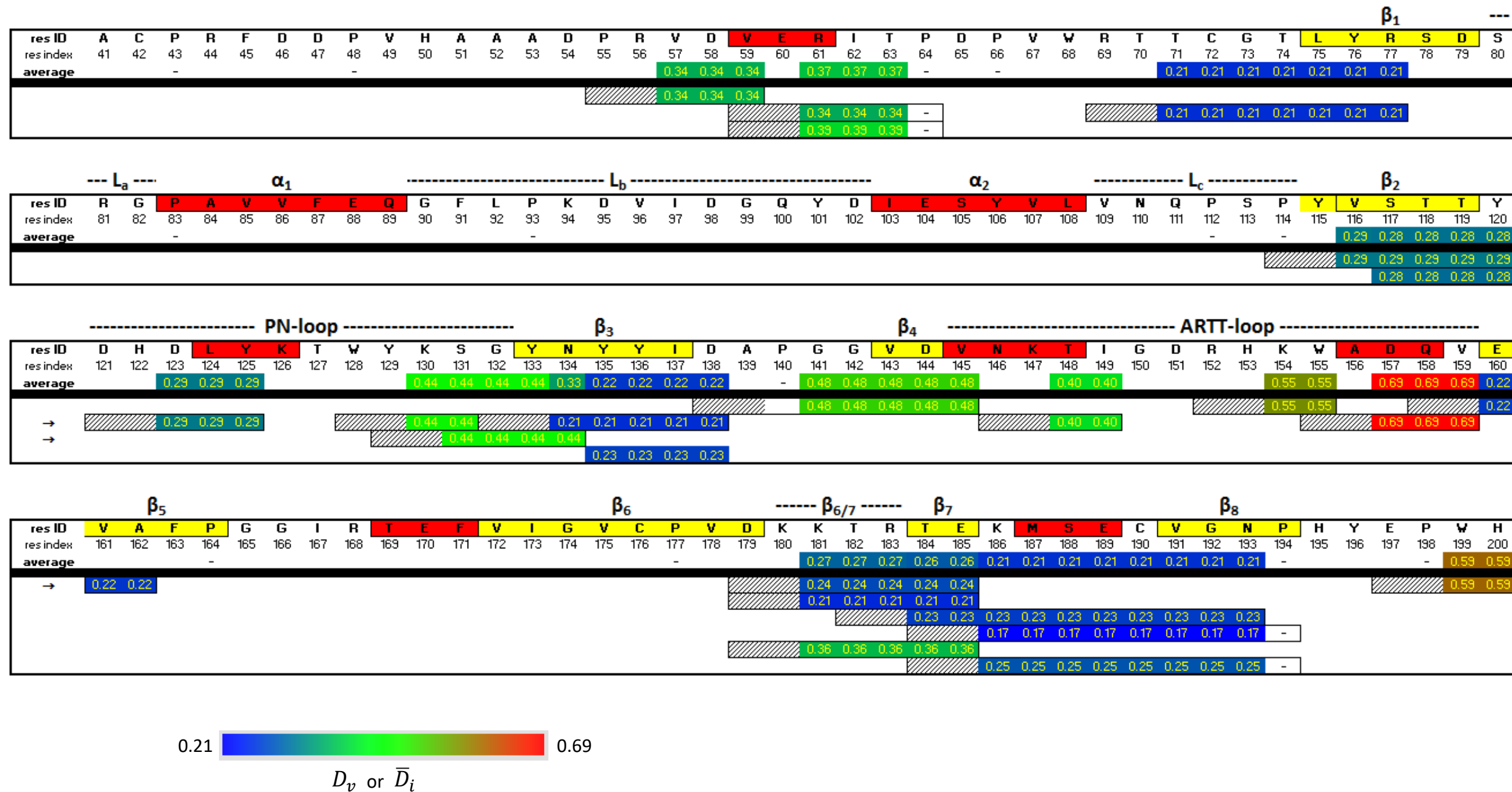

Supplement: S4 Fig — Lower panels. Deuteration level, Dv, of 23 Scabin peptides with lengths between 3 and 12 residues in the presence of DNA. The two C-terminal (striped) and proline (“-”) residues were included in the calculation as indicated in the Materials and Methods. Upper panels. Residue ID, Residue index, and average deuteration level at the residue-level, D-i. In both cases, values spectrally colored from blue (low)–green (medium)–red (high). (PDF) [file pone.0194425.s004.pdf]
